# Humans vs. Robots: Utilization of pointing cues and the mediators of trust in nonverbal communication

**DOI:** 10.1016/j.chbah.2026.100356

**Published:** 2026-07-15

**Authors:** Elizabeth R. Merwin, Jacqueline Hammack, Lindsey Riera-Gomez, Joseph R. Keebler, Erik D. Engeberg, Teresa Wilcox

**Affiliations:** a 777 Glades Road (BS-12, Room 101), Boca Raton, FL, 33431, USA; b 1 Aerospace Blvd, Daytona Beach, FL, 32114, USA

## Abstract

As robots are increasingly acting as communicative agents, it is critical to understand how individuals interpret and utilize their nonverbal information. Pointing gestures are a foundational form of nonverbal communication, yet it remains unclear whether robot-produced pointing is treated as equally communicative as human-produced pointing. The present study examined how pointing gestures were utilized when produced by a Human Hand, a Humanoid Hand, and a Tripod Gripper Hand, and how anticipatory looking related to perceived traits and trust. Eye tracking was used to capture anticipatory visual attention as a measure of cue utilization, alongside post-interaction assessments of trust and perceived agent attributes. Results indicated that participants utilized pointing cues made by the Human Hand and Humanoid Hand, but not by the Tripod Gripper Hand, despite matched cue direction and cue reliability. Perceived trust was not directly associated with whether pointing cues were utilized between agent conditions. Perceived intelligence and likability emerged as the strongest predictors of trust, with lower perceived intelligence mediating the relationship between agent condition and trust for Tripod Gripper Hand relative to the Human Hand. Overall, these findings illustrate the necessity of utilizing behavioral measures with subjective evaluations while demonstrating that trust in nonverbal robotic communication contexts is shaped by evaluative perceptions rather than agent appearance or actions alone. These results offer practical implications for the design of social robots intended to communicate nonverbal information and highlight the importance of designing social robots whose perceived competence and social qualities support their role as effective nonverbal communicators.

## 1. Introduction

The use of social robotics across domains is rapidly expanding. In educational settings, robots serve as tools for teachers in select K-12 schools and have demonstrated success in enhancing student educational outcomes (Benitti, 2012; Di Lieto et al., 2017). They are being used to fill entry-level positions in hospitality, tourism, retail, and food industries to streamline service and reduce business costs (Kang et al., 2023; Kao & Huang, 2023; Ladeira et al., 2023; Wang et al., 2023). Overall, robots serve to make industries run more smoothly by taking over monotonous work and streamlining tasks for increased efficiency (Hagele, 2017). Initially, the design and integration of robots into various settings focused on the services that the robot could provide independently, reducing the workload of humans. As robotic systems become more capable and more deeply integrated into shared workspaces, this role is shifting (Phillips et al., 2011). Rather than acting as standalone tools, robots are increasingly positioned as teammates and communicative partners, expected to coordinate with humans, signal relevant information, and support joint activity. In such contexts, successful interaction depends not only on what a robot does, but on whether its behavior is interpreted as intentional, informative, and worth attending to. Establishing the conditions under which robot behavior is treated as meaningful communication is therefore a central challenge for the design of social robots (Miraglia et al., 2023; Neto et al., 2018; Shrinah et al., 2024).

The rapid expansion of robot use across diverse settings and growing reliance on robots as sources of information is outpacing research regarding the understanding of the factors that influence robot-human communication. This gap is particularly concerning in the development of social robots, where clear and effective communication is essential to a robot’s success as a social agent. This challenge is particularly pronounced for nonverbal communication. Nonverbal cues such as pointing do not explicitly state their meaning but instead require recipients to infer communicative intent (Sauppé & Mutlu, 2014; Tomasello et al., 2007). Whether these cues are utilized depends on expectations about the agent producing them, including perceptions of the agent’s capabilities, credibility, and role in the interaction (Lee & See, 2004; Phillips et al., 2011). The present study addresses this gap by examining how nonverbal pointing gestures are behaviorally utilized when produced by human and robotic agents, and how trust and perceived agent attributes shape the interpretation and use of these cues. Specifically, individuals’ receptivity to nonverbal gestural communication was investigated, focusing on finger pointing, from robotic agents with varying levels of human-likeness and dexterity. Additionally, the perceived attributes of the presented agents are considered as possible mediators between the agent condition and perceived trust.

### 1.1. Robot-human nonverbal communication: the case for pointing

Non-verbal communicative cues, including gestures and non-linguistic vocalizations, are critical to effective and efficient human communication. In certain contexts, they may even surpass verbal communication in importance (Carmichael & Mizrahi, 2023; Norris, 2011). A substantial portion of information in human interactions is conveyed non-verbally, and the absence of such cues can make communication more difficult to interpret and less memorable (Hall et al., 2019; Harrison, 1970; Mehrabian & Ferris, 1967). Integrating non-verbal cues into robotic design enhances human perceptions of robots, making them appear more approachable and interactions more natural. This integration can also lead to improved task performance (Lohse et al., 2014; Salim, 2023; Urakami & Seaborn, 2023). Non-verbal gestural cues are especially valuable in environments where verbal communication is limited, such as noisy industrial settings where robots are often utilized (Sheikholeslami et al., 2017). Furthermore, non-verbal behaviors have been shown to increase task efficiency and support more effective robot-human collaboration, particularly in complex tasks that demand high cognitive load and memory retention (Admoni et al., 2016; Häring et al., 2012).

One particularly salient type of nonverbal cue commonly is the ostensive cue (Miraglia et al., 2023; Sheikholeslami et al., 2017; Shrinah et al., 2024). Ostensive cues are social behaviors, such as direct eye contact and pointing, that signal to the recipient the sender’s intent to communicate and share information with them (Csibra & Gergely, 2009). Humans show sensitivity to ostensive cues from early infancy, prior to acquiring language and extensive social experiences, and they are used universally to communicate the intent to engage in information exchange (Csibra & Gergely, 2006; Liszkowski et al., 2012). Some theorists argue that humans are biologically predisposed to learn through communication facilitated by ostensive signals (Csibra & Gergely, 2009, 2011). These non-verbal cues support communication across diverse contexts, regardless of language background or prior social exposure, making them especially valuable for designing intuitive human-robot interactions (HRIs).

Finger pointing is one of the most widely used and extensively studied ostensive cues in robot-human communication (Alikhani et al., 2020; Das, 2021; Mahmud et al., 2022; Neto et al., 2018). Adults naturally and frequently use pointing gestures, particularly when interacting with less experienced individuals such as infants and young children (Csibra & Gergely, 2011; Liszkowski et al., 2012). This gesture communicates a clear message, “pay attention to this, it is important,” and signals the intent to share information. In most contexts, pointing serves as an efficient means of directing attention toward objects or events, often replacing the need for verbal explanation. Moreover, pointing can enhance verbal communication by clarifying ambiguities or supplementing spoken information (Häring et al., 2012; Lohse et al., 2014; Sugiyama et al., 2007). Crucially, it also facilitates the establishment of *joint attention*, a cognitive mechanism that supports smoother conversational transitions and more effective information exchange (Butterworth, 2004; Tomasello et al., 2007). In summary, finger pointing is a powerful and intuitive non-verbal cue that not only conveys the intent to share knowledge but also creates the shared attentional context necessary for meaningful communication.

A growing body of research supports the efficacy of pointing gestures in the design of social robots (Canuto et al., 2022; Mahmud et al., 2022; Shrinah et al., 2024). Whether observing prerecorded videos or engaging in direct interaction, individuals reliably interpret robotic pointing as a reference to specific objects or locations (Alikhani et al., 2020; Haddadi et al., 2013; Gleeson et al., 2013; Quintero et al., 2015). However, the degree to which robotic pointing is perceived as communicative can vary. Interpretation depends on factors such as the robot’s design features and the context in which the gesture is observed (Ende et al., 2011; Sauppé & Mutlu, 2014). For instance, in industrial pick-and-place tasks (i.e., pick up an object and place it there), the anthropomorphic qualities of a robot do not significantly affect the human interpretation of its pointing gestures. In this context, humans accurately interpret the intent of pointing gestures produced by robots with high, low, or no anthropomorphic features (Alikhani et al., 2020; Ende et al., 2011; Haddadi et al., 2013; Sheikholeslami et al., 2017). Even a simple robotic arm equipped with a directional pointer is sufficient to guide human users in object manipulation tasks. This highlights the robustness and utility of pointing as a communicative tool in human-robot collaboration, regardless of the robot’s physical form.

In contrast, other tasks require more complex robotic hand configurations for effective communication. For example, Sheikholeslami et al. (2017) investigated human interpretation of robotic hand gestures during a collaborative task involving the assembly of a car door panel, a process requiring a sequence of actions on multiple parts. Prior to the experiment, human-human interactions during similar assembly tasks were analyzed to identify commonly used human-inspired communicative gestures, such as index-finger pointing, thumbs up, and the V-sign. The study found that these human-inspired gestures were more accurately recognized and interpreted with greater confidence when performed by a robotic hand equipped with actuating fingers (i.e., fingers that are capable of flexing and extending) as opposed to a robotic hand without actuating fingers. This highlights the communicative advantage of using a five-fingered, posable robotic hand in tasks that demand nuanced gesture interpretation, including index-finger pointing. These findings also suggest that to fully understand the efficacy of pointing gestures in robot-human interaction, researchers must systematically vary robotic hand configurations and compare human interpretations of gestures produced by robotic hands of differing anthropomorphism.

Note that in this research domain, one common experimental approach is to first identify gestures observed in human interactions and confirm that human participants can reliably interpret these gestures in the experimental context under investigation (e.g., Sheikholeslami et al., 2017). Once this is established, the same gestures are programmed into a robotic agent, and human participants’ interpretations of the robot-produced gestures are assessed (Canuto et al., 2022; Deichler et al., 2023; Shrinah et al., 2024). A limitation of this approach is the absence of direct comparisons between robot- and human-produced gestures within the same experimental design. Human control groups, in which participants observe humans performing the same gestures as robots, are rarely included. As a result, while such studies can evaluate how effectively a robot conveys information through a given gesture, they do not assess how this performance compares to the original human gesture from which it was derived. Incorporating direct human-robot gesture comparisons would provide a more comprehensive understanding of the communicative efficiency of robotic gestures. It would also help identify where robotic performance aligns with or diverges from human norms, offering valuable insights for improving gesture design in HRI.

The value of directly comparing robot- and human-produced gestures is exemplified in one of the few studies that implemented such a design. Quintero et al. (2015) assessed adults’ interpretation of identical gestures performed by a human and a robotic agent. In this study, the robot hand was rigid and lacked actuating fingers or digits. Results revealed an 18% increase in gesture misinterpretation when the gestures were produced by the robot as compared to the human agent. The authors attributed this discrepancy to the robot’s non-anthropomorphic design and its inability to point with a straight finger due to under actuation constraints. Although subsequent research has demonstrated that actuating fingers significantly improves gesture recognition (Sheikholeslami et al., 2017), this study highlights a broader issue: certain human gestures, which rely on many degrees of freedom, may not translate effectively to robotic platforms with more limited mechanical capabilities. To advance the field of HRI, it is essential to systematically investigate how specific design features, including finger actuation, impact the interpretation and communicative success of robot-produced gestures, in direct comparison to human gestures.

### 1.2. Perceived agent attributes and trust in nonverbal communication

In addition to understanding what a gesture means, individuals must decide whether the information conveyed by the gesture is worth using. Ostensive cues such as gaze and pointing can effectively direct attention but often require recipients to evaluate if the information being communicated is meaningful, reliable, and relevant to their goals (Caruana et al., 2021). Unlike explicit verbal communication, nonverbal cues often require interpretation by the recipient (Saunderson & Nejat, 2019). As a result, recipients must rely on contextual cues and pre-established expectations about the communicator to determine how much importance to place on the gestures (Alarcon et al., 2023; Kok & Soh, 2020; Schniter et al., 2020). In these circumstances, trust in the sender becomes especially important, as it shapes how nonverbal information is evaluated and whether it is treated as informative guidance or as irrelevant information that should be ignored (Jessup et al., 2025; Kok & Soh, 2020; Robinette et al., 2017).

Trust, in this context, functions as an evaluative filter for communication that influences the recipient’s utilization of nonverbal information. When trust is high, individuals are more likely to accept the information transmitted by the cue, rely on it when making decisions, and use it to guide their attention and behavior (Jessup et al., 2025; Kok & Soh, 2020). When trust is low, the same cues may still be perceived but are more likely to be discounted or ignored (Robinette et al., 2017). This evaluative role of trust is particularly relevant in interactions where communicative intent must be inferred rather than explicitly stated. As robots are increasingly positioned as sources of important information, there is an ever-growing need to understand how humans evaluate and respond to robot-produced nonverbal information.

Trust in robots has been extensively studied and remains a strong predictor of individuals’ future willingness to interact with robots (Hancock et al., 2011, 2023; Hostettler et al., 2023; Schaefer et al., 2016). However, it is important to distinguish between perceived trust, which reflects an individual’s subjective evaluation of an agent, and behavioral trust, or reliance, which reflects whether and how information provided by the agent is utilized (Lee & See, 2004). While closely related, these two constructs do not always align in applied settings. Empirical work has demonstrated dissociations between self-reported trust and reliance on automated aids, highlighting limitations of treating trust as a direct proxy for information use (Dzindolet et al., 2002). Understanding how trust relates to the utilization of nonverbal information requires consideration of both subjective evaluations of the agent and recipient’s observable behavior, something that is commonly overlooked in the current HRI literature.

Individuals may rely on information from an agent (high behavioral trust) despite rating the robot as untrustworthy (low perceived trust), or vice versa, reporting high trust in the agent without relying on the information it provides. In a simulated emergency evacuation scenario, Robinette et al. (2017) exposed participants to a robot that behaved unreliably during the initial familiarization. Participants could report that the robot acted as a bad guide (lower perceived trust in its capabilities) during routine tasks (e.g., leading the participant through a building in a path that was not straightforward), but were still over-whelmingly likely to follow the robot’s directions during the simulated emergency (an indicator of reliance). The dissociation between perceived trust and behavioral reliance suggests that trust judgments cannot be explained by observed performance or subjective trust surveys alone, particularly under conditions of uncertainty.

This pattern reinforces the role of additional factors in trust formation, including perceptual traits associated with robotic agents, which have been shown to be robust predictors of trust (Hancock et al., 2011; Hancock et al., 2023). These perceived traits function as heuristics that guide individuals’ evaluations of an agent’s credibility, competence, and social intent (Christoforakos et al., 2021; Hancock et al., 2011). In turn, these evaluations shape whether nonverbal cues are attended to, interpreted as meaningful, and used to guide behavior, particularly in interactions where communicative intent must be inferred rather than explicitly stated (Kok & Soh, 2020). In human–robot interaction, attributes such as anthropomorphism, animacy, perceived intelligence, and likability have each been shown to influence trust in distinct. Table 1 synthesizes key prior work describing these effects.

### 1.3. Agent expectations and trust

Despite the rapid growth of robotics research and increasing insight into how robots are perceived across contexts, a key limitation of the current literature is the lack of explicit comparisons examining how information from human and robotic agents are evaluated and used when they perform nonverbal pointing tasks. Due to this, it remains unclear whether humans and robots are perceived as equally capable social agents for transmitting nonverbal information, such as pointing cues, or how prior expectations regarding the agent influences the utilization of those cues. Addressing this gap has important implications for the design and integration of robots intended to function as communicative partners. For example, a robot may be designed to nonverbally convey task-relevant information through gestures, yet differences in how humans interpret the robot’s gestures could limit whether those cues are treated as meaningful, ultimately undermining the effectiveness of the interaction despite correct execution of the behavior.

One reason information from humans and robots cannot be assumed to be perceived or interpreted in the same way is the presence of mental models: internal preexisting representations that guide an individual’s expectations about how a system is likely to behave (Rouse & Morris, 1986). Individuals bring prior expectations about an agent’s capabilities, intent, and role into interactions that are not necessarily transferable across human and robotic agents (Kahn et al., 2011; Phillips et al., 2011). In human-human interaction, individuals are assumed to possess shared social intent and contextual understanding and are treated as competent, often knowledgeable communicators (Wolf & Tomasello, 2023). These expectations shape whether gestures are interpreted as intentional communicative signals. As a result, gestures such as pointing may be more effectively interpreted and utilized when produced by humans compared to robots, but there is little research directly comparing this.

According to the computers are social actors (CASA) framework, individuals are likely to apply preexisting human mental models (i.e., their expectations of how a *human* would act in a similar scenario) to a robot they are interacting with (Gambino et al., 2020; Nass & Moon, 2000). For example, if a humanoid-robot were to wave at a person, that person would be likely to assume the robot is greeting them, as that is often the purpose of the gesture when performed another human. There is preliminary research to suggest that the more anthropomorphized the robot, the stronger these human mental models are applied to the robot (Kwon et al., 2016). However, in some contexts, robots are not attributed the human expectations and are perceived to act more as machines. In these contexts, individual’s trust and willingness to have future interactions with the robot are grounded primarily in the perceived competence, reliability, and predictability of the robot rather than any human-like expectations (Lee & See, 2004; Hoff & Bashir, 2014). These differing lenses provide a basis for why behaviors may be interpreted differently depending on whether the gesture source is human or robotic, and the level of human-like social expectations that are applied to the robot.

Such expectation-driven differences are especially consequential in nonverbal communication contexts, where communicative intent must be inferred rather than explicitly stated. Nonverbal cues do not carry explicit instructional content. Instead, their interpretation depends on human assumptions about the communicator’s intent (Hall et al., 2019; Saunderson & Nejat, 2019). As a result, expectations about the agent are shaped by both agent identity and perceived attributes which influence whether a nonverbal cue is interpreted as intentional communication or dismissed as incidental movement (Csibra & Gergely, 2009; Tomasello et al., 2007).

Together, natural pedagogy and the CASA framework offer complementary theoretical explanations of how robot movements are understood and attended to. Natural pedagogy depicts why ostensive cues such as pointing are recognized by humans as communicative acts since humans are predisposed to interpret them as signals of intent to share information (Csibra & Gergely, 2009). The CASA framework extends this finding past human-human interaction to explain that the extent to which those cues are treated as credible and worth attending to depends on the mental models individuals apply to the robot producing them (Nass & Moon, 2000). When a robot is attributed human social norms and expectations, its behaviors are more likely to be interpreted as intentional and informative, while a robot being perceived as mainly a machine would be less likely to elicit the same expectations. Agent identity and its perceived attributes may guide the extent of expectations about a robot’s communicative intent, for example, higher perceived anthropomorphism may increase the likelihood that a robot’s nonverbal actions are interpreted intentionally produced and socially meaningful (Kiesler et al., 2008; Złotowski et al., 2016).

This distinction between intentional and ambiguous nonverbal cues becomes particularly salient when an agent’s reliability (i.e., the extent to which the robot’s behaviors are consistently accurate) is not perfect (Lee & See, 2004; Salem et al., 2015). Under conditions of imperfect reliability, individuals may show continued, but cautious reliance on robots, neither fully discounting the agent’s information nor accepting it uncritically (Lee & See, 2004; Parasuraman & Riley, 1997; Robinette et al., 2017). In these circumstances, trust becomes especially relevant, as it guides decisions about whether nonverbal cues should be used to inform behavior (Kok & Soh, 2020). When the credibility of the information source is uncertain, individuals cannot rely solely on the presence of a cue but must decide whether the information conveyed by that cue is worth using (e.g., whether a pointing gesture should be followed or ignored). Based upon the agent type, human or robot, differences in baseline expectations and perceived attributes may lead to the same nonverbal behavior being relied upon differently. Understanding how reliance and trust interact is critical for evaluating how and why nonverbal cues are utilized across different agent types and motivates the questions addressed in the present study.

### 1.4. Present study

Prior work demonstrates that nonverbal cues, especially pointing, can effectively direct attention and support information exchange (Csibra & Gergely, 2009), and that trust in the communicator plays a critical role in determining whether information from an agent is evaluated and used (Jessup et al., 2025; Kok & Soh, 2020). Perceptual factors such as anthropomorphism, animacy, intelligence, and likability shape trust and expectations about the agents (Christoforakos et al., 2021; Hancock et al., 2011; McKenna et al., 2024), however, few studies directly compare human and robotic agents performing similar nonverbal behaviors to explore how differences in perception, trust, and utilization of nonverbal cues emerge. This limits the conclusions that can be drawn about whether robots are treated as communicative partners in the same way as humans. In addition, trust and agent perception are often evaluated independently from behavioral indicators of information use, making it unclear how subjective evaluations relate to actual reliance on nonverbal cues. The present study addresses these gaps by directly comparing human and robotic pointing gestures and integrating measures of visual attention and reliance on nonverbal cues with subjective assessments of trust and agent attributes.

This study takes a novel approach to examining individuals’ receptivity to nonverbal information from human and multiple robotic agents by directly comparing pointing gestures and analyzing dwell time and perceptions of the agents. This approach provides a more comprehensive understanding of how nonverbal cues are interpreted and used across human and robotic communicators and addresses the following research questions:

RQ1: To what extent do participants utilize pointing gestures as communicative cues, through predictive visual attention, and does this differ across human and robot agents?
RQ2: Is there a relationship between the utilization of nonverbal cues and subjective trust, and does this differ by agent?
RQ3: How does the agent type influence perceived attributes, including anthropomorphism, animacy, likability, and perceived intelligence?
RQ4: To what extent are differences in trust between agent types explained by the agent type and the perceived agent attributes?

## 2. Method

### 2.1. Participants

Participants included 106 individuals (79 female, average age 19.3 years (*SD* = 0.2); see Table 2 for full demographics) from a small southeast university in the United States of America. The experiment was approved by the university’s Institutional Review Board (IRB # 2406217) and recruitment of participants was conducted through the university’s research participant pool system. All participants gave consent prior to participating, had normal or corrected-to-normal vision, reported no colorblindness, and received either course credit or extra credit for their participation. An additional two participants completed the experiment but were not included in the sample due to incomplete data collection.

### 2.2. Procedure

Upon arrival at the testing space, individuals consented to the experimental procedure. Participants were then randomly assigned to one of the three agent conditions: Human Hand (HUH), Humanoid Hand (HH), or Tripod Gripper Hand (TGH). All participants were tested using the same procedure but were only shown the specific agent they were assigned to. Participants completed a demographics questionnaire on a computer, as well as the Mini-IPIP (Donnellan et al., 2006) and the Interpersonal Trust Scale (Rotter, 1967). The Mini-IPIP and the Interpersonal Trust Scale were collected as part of a larger project and were not used in the analyses conducted here. After completing the pre-experimental surveys, participants were led into a separate testing room where they viewed video stimuli of their agent (detailed in the Stimuli and Apparatus section). After watching the video stimuli, participants completed two surveys to evaluate their perception of the agent: the Godspeed questionnaire and the Trust in Automation scale (see Appendix A for the surveys used). The Godspeed questionnaire assessed participants’ perceived anthropomorphism, animacy, likability, and intelligence of the agent (Bartneck et al., 2008). The Trust in Automation scale assessed participants’ perceived trust in the agent (Jian et al., 2000). After these surveys were completed, participants were thanked for their time and provided with a debrief on the nature of the study. Participants were then encouraged to ask any questions they had related to the experiment, were asked not to share the details of the experiment with others and were compensated.

### 2.3. Measures

#### 2.3.1. Godspeed questionnaire (Bartneck et al, 2008)

The Godspeed questionnaire is commonly used to assess human perceptions of agents across several key dimensions: anthropomorphism, animacy, likability, intelligence, and perceived safety (Appendix A1). Each dimension is measured using semantic differential items (e.g., “*fake-natural*,” “*dead-alive*,” “*unfriendly-friendly*,” “*incompetent-competent*”), which participants rated on a 5-point Likert scale with the items listed so that higher scores indicate the more positive/humanlike perception. Due to the experimental design, in which the participants never directly interacted with the agent seen (see section 2.4 Experimental Design for more details), the perceived safety subscale was not utilized. The Godspeed questionnaire has been shown to possess acceptable internal consistency across studies, with Cronbach’s alpha values for subscales typically ranging from .70 to .90 (Bartneck et al., 2008; Bartneck, 2023). External validity has been supported by strong correlations with observed user behaviors, such as willingness to interact with, follow instructions from, or spend time with a robot (Bartneck et al., 2008; Bartneck, 2023). Composite scores for participant responses were computed in SPSS for each of the subscales measured: anthropomorphism (Cronbach’s alpha = .76), animacy (Cronbach’s alpha = .74), likability (Cronbach’s alpha = .86), and intelligence (Cronbach’s alpha = .77).

#### 2.3.2. Trust in Automation (Jian et al., 2000)

Participants completed the Trust in Automation scale, a heavily utilized measure to assess subjective trust in automated systems (Jian et al., 2000). Example items include judgments of system reliability (e.g., “*I can rely on this automated system to act consistently*”) or potential risks (e.g., “*The system’s actions will have a harmful or injurious outcome*”), rated on Likert scales of 1-7, with 1 representing that the participant strongly disagreed with the statement and 7 being they strongly agreed with the statement. The scale has demonstrated good internal consistency in prior validation studies (Gutzwiller et al., 2025; Jian et al., 2000; Kohn et al., 2021 ). For this study design, survey items that contained the word “system” were replaced with the word “arm,” as is often done with this questionnaire (Gutzwiller et al., 2025). Although Gutzwiller and colleagues (2025) recommended against modifications to the survey items for validity concerns, the nature of the agent presentation used in this study resulted in participant confusion during pilot testing. Some participants misinterpreted the stage setup as the “system” instead of the agent in the video for the Human Hand agent condition. This confusion necessitated a neutral word to capture the actual agent being seen, and “system” was replaced with “arm” to clarify exactly what the participants were supposed to reference for the questionnaire. Items were reverse scored as needed based on the scale’s wording, and the overall threshold for internal reliability for the scale in our sample was exceeded (Cronbach’s alpha = .83).

#### 2.3.3. Visual attention

Anticipatory visual attention was used as a behavioral measure of cue utilization (Stotler, 2021). Dwell time was defined as the summed duration of all fixations within a predefined area of interest during a specified phase of the trial, recorded using the SR Research EyeLink 1000 Plus eye tracking system (SR Research Ltd, 2011). Fixations were identified based on a velocity threshold of 30°/s and a minimum duration of 100 ms. Dwell time to the target AOI during the Prediction phase served as the primary measure of anticipatory looking. Cue utilization was operationalized as the difference in mean dwell time to the target AOI between cued and control trials, isolating anticipatory attention attributable to the agent’s pointing gesture rather than individual differences in general looking behavior.

### 2.4. Stimuli and Apparatus

The test video stimuli were presented in a dimly lit room on a 47.5 × 26.5 cm monitor. During the experiment, participants’ gazes were tracked using the SR Research Eyelink 1000 Plus remote Eye tracking system (SR Research Ltd, 2011). The participants sat in a 45 cm high straight-back chair placed 60-70 cm from the monitor. The Eyelink 1000 eye tracking camera was positioned on a stand 10 cm below the monitor, which was 90 cm off the ground. Participants were instructed to remain still during the experiment, and breaks were given to ensure focus and avoid eye strain. Participants watched a 5-point calibration sequence in which a blue shape appeared on a white background in five different locations to obtain accurate measurements of where the participants were looking. Calibrations ensured quality data collection and were given at the beginning of the experiment and after each break (Holmqvist et al., 2011).

### 2.5. Experimental design

Participants were randomly assigned to one of three agent conditions: the Human Hand agent (HUH), the Humanoid Hand (HH), or the Tripod Gripper Hand (TGH) (see Fig. 1). Each visible portion of the agent consisted of the arm and hand that extended from behind a sectioned white curtain. All agents were covered with a neon green sleeve that extended past the elbow. The sleeves were used to increase the saliency of the agent against the background of the stage and to reduce surface-level visual differences across conditions, such as color, texture, reflectance, and local visual salience. This choice was intended to support comparison of cue utilization across agent types under visually controlled conditions. Similar external coverings have been used in prior HRI gesture studies involving human and robotic agents (Gleeson et al., 2013; Haddadi et al., 2013), although the present study used the sleeves specifically to reduce uncontrolled surface-level differences across conditions (Carter & Luke, 2020). The sleeves did not eliminate the morphologically diagnostic features of the agents.

The Human Hand and the Humanoid Hands both had five fingers that moved independently, while the Tripod Gripper Hand had three pincer-like fingers at the end of its hand (see section 2.5 for a more detailed explanation on the differences in agent movement and appearance). For the Human Hand and Humanoid Hand, the cue was produced through an index-finger point. For the Tripod Gripper Hand, the cue was produced through the orientation of the whole gripper toward the target box due to the hand’s lower degrees of freedom. Recent eye-tracking work demonstrates that adults attend to index-finger, other-finger, and whole-hand points toward objects, with no statistically significant differences in orientation time across these pointing forms (Ger et al., 2024).

In the test trial videos, participants viewed their assigned agent performing a three-phase series of events (see Supplemental Materials for the videos used). Each video had 1) an Action phase, in which the agent would execute a pointing gesture, 2) a Prediction phase, which served as a waiting period, and 3) an Event phase, in which a feathered wand would appear from one of two toy boxes (Fig. 2). The Action phase lasted 15 seconds, the Prediction phases lasted 3 seconds, and the Event phase lasted 2 seconds, meaning every trial was 20 seconds total. All participants viewed three different trial types: two cued and one control. The order in which the three trial types were seen was counterbalanced and randomized. Cued trials were either congruent or incongruent. In the cued congruent trials, the agent pointed at one of the two boxes during the Action phase, and the feathered wand appeared from that same cued box in the Event phase. The cued incongruent trials differed in one way from the congruent trials: the feathered wand in the Event phase appeared from the box that was not cued (did not receive a point). In a control trial, the agent pointed straight down during the Action phase, and the feathered wand randomly appeared from one of the two boxes during the Event phase. All videos were recorded in a controlled laboratory environment against the neutral white backdrop, with consistent lighting and a fixed camera position across the agent conditions to minimize variation between stimuli. See Fig. 2 for a flow map of the phases of each trial type.

Of primary importance to the research questions were the dwell times to the two boxes during the Prediction phase, where participants saw only the two boxes, with no agent or toy present. If participants were receptive to information provided by the agent in the Action phase (i.e., they interpreted the finger-point as having communicative intent), then in the Prediction phase, they should have looked longer at the box to which the finger had cued than to the other box, indicating anticipatory looking (Stotler, 2021). In contrast, in the control trials where the gesture seen in the action phase did not specify a location, participants should have looked relatively equally at the two boxes in the Prediction phase.

In each of the phases, there were three Areas of Interest (AOI), depicted as the white boxes in Fig. 2. The three AOIs were predefined rectangles drawn over the areas where the stimuli were present. The left and right rectangles were equally sized areas (355288[px]) and encompassed the box and the area in which the toy appeared and moved. The middle box surrounded the arm and the entire area in which the arm moved (438592[px]). The AOIs were kept constant across the phases, trial types, and the three agent conditions. The center AOI was the middle box and encompassed the space in which the agent moved. The target AOI was assigned as the box that was pointed at by the agent in the Action phase. In the case of the control trials, one of the two boxes was randomly assigned as the target box. For all trials, the nontarget AOI was the box not assigned as the target AOI.

Participants viewed a total of 48 test trials in three different pseudo-randomized blocks. Trials were broken into three separate blocks (Block 1 with trials 1-16, Block 2 with trials 17-32, and Block 3 with trials 33-48) to provide breaks in the experiment to avoid fatigue. The 48-trial structure was determined through pilot testing, determined by balancing the length of the experiment with the need for adequate statistical power. The combination of these determined that 16 trials per block would produce sufficient observations for stable individual-level dwell time estimates (Carter & Luke, 2020). Blocking the trials also allowed us to assess the extent to which gaze behavior changed over the course of the experiment. The first trial type that participants viewed (congruent, incongruent, or control) was randomized and counterbalanced across participants. Any trials in which the participants were not watching the monitor, detected either through the Eyelink 1000 software or through experimenter notes, were excluded from the analyses.

### 2.6. Robotic hands and arm

In order to perform the robotic gestures in a controlled fashion, each of the robot hands from Fig. 3 was mounted on the same UR5 robotic arm (Universal Robots, Denmark). The Tripod Gripper Hand condition used the Motion Control Hand (Motion Control, Salt Lake City, USA), which had three digits and a single degree of freedom (DOF) that opened and closed the hand in a tripod grip (Feix et al., 2015). The index and middle fingers were mechanically coupled to the thumb by a 4-bar mechanism so that all digits either opened or closed together in unison (Engeberg et al., 2008). In contrast, the Humanoid Hand condition used the i-limb hand, with four fingers and a thumb (Kent et al., 2014). The i-limb had 10 passive DOFs and 6 active DOFs. The thumb had two motors near where the human carpometacarpal joints are located that produce movements similar to circumduction and abduction/adduction or flexion/extension. Each finger had one motor that drove flexion/extension of the metacarpophalangeal joint, with the corresponding proximal interphalangeal joint passively driven via a Kevlar tendon. Previously, the i-limb had been used to explore the impact that simulated malfunctions had upon human trust of the robot (Abd et al., 2019).

The three-fingered Motion Control hand (Tripod Gripper Hand) had the ability to point; however, its motions were produced by a single actuator that could not mimic human hand motions very accurately. Due to these mechanical constraints, the Tripod Gripper Hand produced a whole-hand directional cue using its three-digit pincer configuration rather than an index-finger point. Thus, the gestures of the two robotic hands have two very distinct manipulator morphologies that differ in degrees of human-likeness and dexterity. This provided a large disparity between manipulator capabilities and anthropomorphism, ranging from a dexterous manipulator that closely resembles a human hand (i-limb; Humanoid Hand) and a moderately sophisticated three-fingered hand (Motion Control Hand; Tripod Gripper Hand). It should be noted that dexterity and hand morphology co-varied across agent conditions and were not independently manipulated. As such, dexterity is treated here as a descriptive feature of the agents rather than as an experimentally isolated variable. These robots varied in dexterity and human-like appearance as do many robots utilized in real-life situations.

### 2.7. Data preparation and analysis plan

Demographic data were collected and numerically coded for analysis in SPSS to check for variation based on factors such as gender and race. Eye tracking data were sorted and exported through the Dataviewer software package provided by Eyelink. Average dwell time for each AOI was calculated by the Dataviewer software and was defined as the summed duration across all fixations made in the AOI during the predefined set times. Fixations were identified by the EyeLink algorithm based on a velocity threshold of 30°/s and a minimum duration of 100 ms (SR Research Ltd, 2011). Any fixations that were flagged as extreme outliers were substituted in SPSS through winsorizing (Kennedy et al., 1992) by assigning identified extreme outliers the next highest or lowest value in the dataset. These were computed through finding the 5th and 95th percentiles of the variable and substituting any outliers that were lower or higher than that percentile. Average dwell times were calculated for each phase, trial type, and block of the experiment.

A series of a priori power analyses were performed using G*Power 3.1 to ensure that the planned statistical tests were adequately powered (Faul et al., 2009). To address RQ1, for eye tracking analyses in the Hand and Choice phases, the design included one between-subjects factor (agent condition with 3 levels: HUH, HH, TGH) and three within-subjects factors: Trial Type (point, control), Area of Interest (target, nontarget, center), and Block (Block 1, Block 2, Block 3). The repeated-measures structure was approximated as a mixed design with 18 measurements per participant. Results indicated that a minimum of 21 participants were required to achieve 95% power to detect medium effects (Cohen’s f = 0.25, α = .05). For the Event phase, which included three Trial Type levels (congruent, incongruent, control), 15 participants were required to achieve equivalent power. Both requirements were exceeded in the final sample.

For RQ2, to explore the relationship between reliance on nonverbal agent cues and participant trust scores, cue utilization was calculated for each participant as the difference between mean dwell time to the target AOI in cued and control trials during the Prediction phase. This approach allows for the examination of each participants’ utilization of the agent’s nonverbal cues by measuring how visual attention to the target differed from when it was cued to when it was not cued. A multiple linear regression with cue utilization as the dependent variable, and subjective trust with agent condition (dummy-coded, with Human Hand as the reference condition) acting as predictors was planned. The final model included three predictors (cue utilization, Humanoid Hand, and Tripod Gripper Hand) and G*Power revealed that a minimum of 54 participants were required to achieve 95% power to detect medium effect (Cohen’s f^2^ = 0.15, α = .05), which the final sample exceeded.

RQ3, regarding the perceived agent attributes were analyzed using a one-way MANOVA with agent condition as the independent variable and the Godspeed subscales (anthropomorphism, animacy, likability, intelligence) as dependent variables. The test indicated that a total sample size of 75 participants would be required to achieve 95% power to detect overall group differences assuming a medium effect size (Cohen’s f = 0.25) and a significance criterion of α = .05. The final obtained sample exceeded this requirement.

Finally, for RQ4 to conduct a power analysis for the parallel mediation model that examined whether perceptions of anthropomorphism, animacy, likability, and intelligence mediated the effect of agent condition on trust, the power was approximated using a multiple regression framework consistent with prior literature recommendations (Fritz & MacKinnon, 2007). This framework provided recommended sample sizes for detecting indirect effects based on the magnitude of model pathways. Since the expected effects fall in the small-to-medium range, Fritz and MacKinnon’s (2007) simulation work indicated that samples of 30-60 participants would provide adequate power. In addition to this, an a priori power analysis was conducted with G*Power modeling trust being predicted from five predictors (agent condition and the perceived godspeed attributes), which indicated that the final sample should have 92 participants to achieve 80% power to detect a medium effect (Cohen’s f^2^ = 0.15) at a significance criterion of α = .05. The final sample size exceeded both recommendations. The 80% power criterion applied to the GPower approximation for RQ4 reflects standard practice for regression-based power estimation in mediation contexts, where the complexity of indirect effect pathways makes 95% power targets difficult to specify precisely a priori (Fritz & MacKinnon, 2007).

## 3. Results

The following analyses were conducted to address the four research questions outlined above. Results are presented in a sequence that progresses from eye tracking indicators of communicative cue utilization to the relationship between reliance and trust, and finally to the subjective perceptual factors underlying trust judgments across agent types.

### 3.1. Eye tracking data analyses

In order to address RQ1, visual attention was examined across the Action, Prediction, and Event phases of the task to assess how participants responded to agent pointing behavior over time. Visual attention was defined as the average dwell time to predefined areas of interest across phases and it was compared across the Human Hand, Humanoid Hand, and Tripod Gripper Hand conditions. The Action phase was included to evaluate whether overall attentional allocation differed between the agents when the agent was visible on screen, independent of predictive demands. The Prediction phase served as the primary test of cue utilization, assessing whether participants allocated anticipatory attention to the cued location prior to object appearance. The Event phase was included to examine whether attention to the appearing object differed as a function of agent condition. Since the Action, Prediction, and Event phases served different purposes, statistical follow up procedures differed to align with the confirmatory versus exploratory nature of the setup. For the Action and Event phases, they involved exploratory follow up analyses of higher-order interactions without any specific a priori predictions. In such, post-hoc comparisons were evaluated using the Benjamini-Hochberg false discovery rate procedure (Benjamini & Hochberg, 1995), implemented using ranked p-values and corresponding critical value thresholds to control for multiple comparisons. The Prediction phase, however, was hypothesis driven and focused on planned comparisons testing whether participants allocated greater anticipatory attention to the cued location. These planned contrasts were evaluated using least significant difference tests. This approach ensured that statistical inferences were appropriately matched to the theoretical role of each phase in addressing RQ1.

#### 3.1.1. Preliminary analyses

Preliminary analyses were conducted to examine the effect of gender on participants’ attention to and interpretation of human and robot pointing and are included in Appendix B. To maintain sufficient statistical power, the data were averaged across blocks for the gender analyses. A 3 (Agent: HUH, HH, TGH) × 3 (AOI: center, nontarget, target) × 2 (Trial Type: cued, control) × 2 (Gender: female, male) mixed-model repeated measures analysis of variance (RM ANOVA) was performed on average dwell times (in milliseconds) during the Action phase of the test trials. Agent condition and gender were treated as between-subjects factors, while AOI and trial type were within-subjects factors. No significant main effects or interactions involving gender were found (see Appendix B1).

A second RM ANOVA using the same design was conducted on average dwell times during the prediction phase. Again, no significant main effects or interactions involving gender were observed (see Appendix B2). Given the imbalanced sample sizes for females (n = 79) and males (n = 27), these results should be interpreted with caution. The gender distribution reflects the demographic composition of the subject pools from which participants were recruited.

#### 3.1.2. Action phase

A 3 (Agent: HUH, HH, TGH) × 3 (AOI: center, nontarget, target) × 2 (Trial Type: cued, control) × 3 (Block: Block 1, Block 2, Block 3) mixed-model RM ANOVA was conducted on average dwell times (in milliseconds). Agent condition was treated as a between-subjects factor, while AOI, trial type, and block were within-subjects factors. Descriptive statistics for average dwell times during the action phase are presented in Table 3, and the results of the RM ANOVA are reported in Table 4. Follow-up analyses for higher-order interactions were conducted using the Benjamini-Hochberg false discovery rate (FDR) procedure (Benjamini & Hochberg, 1995). Within each family of post-hoc comparisons, *p*-values were rank-ordered and evaluated against BH critical values ((*i/m*)·α). For transparency, both the raw p-values and the corresponding BH critical values are reported. Here, *q* denotes the BH critical value associated with each ranked comparison rather than an adjusted *p*-value. Effects were interpreted as statistically significant when *p* < *q*.

There were significant main effects of Block and AOI, as well as a significant Block × AOI interaction (see Table 4). To follow up the interaction, paired comparisons were conducted using estimated marginal means, and p-values were assessed using the Benjamini–Hochberg procedure (Benjamini & Hochberg, 1995). Dwell times to the center AOI decreased significantly across blocks, from Block 1 (*M* = 10,646.54, *SE* = 163.07) to Block 2 (*M* = 10,486.10, *SE* = 189.54), *p* = .004, *q* = .006, and again from Block 2 to Block 3 (*M* = 10,218.39, *SE* = 179.71), *p* = .007, *q* = .007. No significant block differences were observed for the target box (Block 1: *M* = 840.88, *SE* = 52.36; Block 2: *M* = 838.97, *SE* = 62.67; Block 3: *M* = 849.73, *SE* = 55.80), *p*s = .928 and .747, or the non-target box (Block 1: *M* = 153.11, *SE* = 12.80; Block 2: *M* = 142.39, *SE* = 15.86; Block 3: *M* = 151.96, *SE* = 12.59), *p*s = .19 and .454.

A significant Trial Type × AOI interaction also emerged (see Table 4; Fig. 4). In cued trials, participants looked longer at the target box (*M* = 1520.31, *SE* = 106.58) than at the non-target box (*M* = 156.59, *SE* = 12.71), *p* < .001, *q* = .008. In control trials, looking times to the target (*M* = 166.08, *SE* = 17.58) and non-target (*M* = 141.71, *SE* = 14.84) boxes did not differ significantly, *p* = .017, *q* = .0125. Participants also looked significantly longer at the center AOI in control trials (*M* = 11,180.68, *SE* = 181.71) than in cued trials (*M* = 9720.00, *SE* = 179.71), *p* < .001, *q* = .01. Together, these findings show that when the finger point was directed downwards participants attended primarily to the center AOI; when the finger was pointing towards a box the Action phase, participants split their attentional allocation between the center AOI and the cued AOI, decreasing dwell time to the center AOI.

The ANOVA further revealed a significant Trial Type × AOI × Agent interaction (see Table 4). For experimental trials, an AOI × Agent interaction was found, *F*(4, 206) = 3.80, *p* = .016, ηp^2^ = .069 (equal variances not assumed), but none of the follow up comparisons survived FDR correction. Dwell times to the center AOI did not differ significantly between Human Hand (*M* = 9710.40, *SE* = 312.71), Humanoid Hand (*M* = 9199.50, *SE* = 312.71), and Tripod Gripper Hand (*M* = 10,250.13, *SE* = 308.34), *p*s = .251 and .222, respectively. Similarly, target AOI dwell times did not differ significantly between Human Hand (*M* = 1480.54, *SE* = 185.47), Humanoid Hand (*M* = 1876.80, *SE* = 185.47), and Tripod Gripper Hand (*M* = 1203.58, *SE* = 182.88), *p*s = .134 and .290. For the non target AOI, the Human Hand versus Humanoid Hand comparison (Human: *M* = 139.85, *SE* = 22.11; Humanoid: *M* = 116.74, *SE* = 22.12) was nonsignificant, *p* = .462, and although the Human Hand versus Tripod Gripper Hand comparison (*M* = 213.20, *SE* = 22.81) yielded *p* = .020, it did not reach significance after correction (*q* = .008).

For control trials, the main effect of AOI was significant, *F*(2, 206) = 3488.11, *p <* .001, ηp^2^ = .971, with longer looking to the center AOI (*M* = 11,180.68, *SE* = 181.71) than to the target (*M* = 166.08, *SE* = 17.58) or non target (*M* = 141.71, *SE* = 14.84) AOIs (both *p*s < .001). Neither the main effect of Agent, *F*(2, 103) = 1.68, *p* = .191, nor the Agent × AOI interaction, *F*(2, 103) = 0.92, *p* = .404, was significant. Because the Trial Type × AOI × Agent interaction had a small effect size and follow up tests did not survive FDR correction, these results are not interpreted further.

#### 3.1.3. Prediction phase

A 3 (Agent: HUH, HH, TGH) × 3 (AOI: center, nontarget, target) × 2 (Trial Type: cued, control) × 3 (Block: Block 1, Block 2, Block 3) RM ANOVA was conducted on mean dwell times (in milliseconds) during the Prediction phase. Agent condition was treated as a between-subjects factor, while AOI, trial type, and block were within-subjects factors. Descriptive statistics for mean looking times during the prediction phase are presented in Table 5, and the results of the analysis are reported in Table 6.

The ANOVA revealed a significant main effect of AOI, and significant interactions for AOI × Trial Type and Trial Type X Agent, (Table 6; Fig. 5). All of which were qualified by a significant Trial Type × AOI × Agent interaction with a medium-large effect size, as predicted. Planned contrasts using the Least Significant Difference test to control for multiple comparisons showed the following patterns.

1. Human Hand. In the cued condition, participants looked significantly longer at the target box (*M* = 496.49, *SE* = 74.67) than the non-target box (*M* = 338.40, *SE* = 46.28), *p* = .03. In the control condition, looking times did not differ between the target (*M* = 225.29, *SE* = 38.77) and non-target (*M* = 239.79, *SE* = 38.77) boxes, *p* = .613.
2. Humanoid Hand. In the cued condition, participants again looked significantly longer at the target box (*M* = 359.30, *SE* = 74.67) than the non-target box (*M* = 209.07, *SE* = 46.21), *p* = .039. In the control condition, no significant difference emerged between the target (*M* = 243.38, *SE* = 40.66) and non-target (*M* = 204.98, *SE* = 38.77) boxes, *p* = .183.
3. Tripod Gripper Hand. Looking times did not differ significantly between target and non-target boxes in either the cued condition (target: *M* = 435.19, *SE* = 73.62; non-target: *M* = 359.11, *SE* = 45.63), *p* = .286, or the control condition (target: *M* = 290.35, *SE* = 40.09; non-target: *M* = 295.88, *SE* = 38.23), *p* = .845.

Overall, these results suggest that participants who viewed the Human Hand or Humanoid Hands used the pointing gesture to anticipate where the wand would later appear, indicating that they interpreted the gesture as being communicative. In contrast, participants who viewed the Tripod Gripper Hand did not use the pointing gesture to guide their predictions, suggesting that they did not interpret its movements as conveying intentional information.

#### 3.1.4. Event Phase

The Event phase tested whether attention to the appearing object varied by agent condition. In the Action and Prediction phases, trials were either cued (i.e., the finger pointed to one of the boxes) or control (i.e., the finger pointed downward between boxes). In the Event phase, trials were congruent (i.e., the wand appeared in the cued box previously pointed to), incongruent (i.e., the wand appeared in the opposite box, meaning the one not cued), or control (i.e., the wand randomly appeared).

A 3 (Agent: HUH, HH, TGH) × 3 (AOI: center, nontarget, target) × 3 (Trial Type: congruent, incongruent, control) × 3 (Block: Block 1, Block 2, Block 3) RM ANOVA was conducted on mean dwell times. Agent was a between-subjects factor; AOI, trial type, and block were within-subjects factors. Descriptive statistics for the Event phase appear in Table 7, and ANOVA results are presented in Table 8. Follow-up analyses for higher-order interactions were conducted using the Benjamini–Hochberg false discovery rate (FDR) procedure (Benjamini & Hochberg, 1995). Within each family of post-hoc comparisons, *p*-values were rank-ordered and evaluated against BH critical values ((*i/m*)·α). For transparency, both the raw p-values and the corresponding BH critical values are reported. Here, *q* denotes the BH critical value associated with each ranked comparison rather than an adjusted *p*-value. Effects were interpreted as statistically significant when *p* < *q*.

The ANOVA revealed significant main effects of block, trial type, and AOI (see Table 8). The following significant two-way interactions were also obtained: Block x Trial Type, Block x AOI, and Trial Type x AOI.

All effects were qualified by a significant Block × Trial Type × AOI interaction (Table 8). In congruent trials, looking times to the cued box changed across blocks, significantly decreasing from Block 1 (*M* = 1340.14, *SE* = 18.29) to Block 2 (*M* = 1179.59, *SE* = 33.52), *p* < .001, *q* = .006, and then significantly increasing from Block 2 to Block 3 (*M* = 1352.99, *SE* = 19.36), *p* < .001, *q* = .007, whereas looking times to the non-cued box did not differ significantly (Block 1: *M* = 51.58, *SE* = 3.21; Block 2: *M* = 49.11, *SE* = 4.76; *p* = .443; Block 2 vs. Block 3: *M* = 40.51, *SE* = 3.32; *p* = .035, *q* = .0083—not significant after correction). For incongruent trials, looking times to the cued box showed no significant block differences, with Block 1 (*M* = 72.35, *SE* = 4.81) not differing from Block 2 (*M* = 64.61, *SE* = 5.43), *p* = .035, *q* = .006 (not significant after correction), or Block 2 from Block 3 (*M* = 67.21, *SE* = 6.36), *p* = .677; likewise, non-cued box looking times did not differ (Block 1: *M* = 1302.30, *SE* = 17.62; Block 2: *M* = 1314.43, *SE* = 21.18; *p* = .232; Block 2 vs. Block 3: *M* = 1290.87, *SE* = 19.44; *p* = .17). Control trials also showed no significant block wise differences for the cued box (Block 1: *M* = 1354.65, *SE* = 26.90; Block 2: *M* = 1376.37, *SE* = 34.41; *p* = .321; Block 2 vs. Block 3: *M* = 1330.05, *SE* = 32.12; *p* = .153) or for the non-cued box (Block 1: *M* = 72.35, *SE* = 4.81; Block 2: *M* = 64.61, *SE* = 5.43; *p* = .007, *q* = .006 - not significant after correction; Block 2 vs. Block 3: *M* = 52.63, *SE* = 6.40; *p* = .020, *q* = .007 - not significant after correction).

#### 3.1.5. Relationship between anticipatory looking and subjective trust

To address RQ2 a linear regression was conducted to examine whether subjective trust was associated with utilization of nonverbal cues during the Prediction phase. Cue utilization was calculated as the difference between the mean dwell time to the target AOI in cued trials and control trials to account for individual differences in looking behavior and to isolate anticipatory looking guided by the agent cues. Agent condition was dummy coded with the Human Hand serving as the reference group; and was included as a covariate. The overall model was not significant *F*(3, 102) = 1.04, *p* > .05, *R*^2^ = .03. Subjective trust ratings were not statistically associated with cue utilization, *B* = −18.73, *SE* = 43.59, *t* = −0.43, *p* > .05, indicating that trust did not covary with the extent to which participants used pointing cues to guide anticipatory attention.

### 3.2. Perception of agents

Participants’ subjective assessments of the agents were examined to explore how perceptual factors relate to trust judgments when participants only interacted with one of the agent types. This allowed for a controlled approach to compare agents of different human-likeness and dexterity to the Human Hand condition when all else was constant. Table 9 presents the means and standard errors for the measured Godspeed subscales and perception of trust in the different agents. The following subsections aim to address RQ3 and RQ4.

#### 3.2.1. Differences in perceived agent attributes

To address RQ3, a one-way multivariate analysis of variance (MANOVA) was conducted to examine whether perceptions of the agents (HUH, HH, TGH) differed across the Godspeed subscales (anthropomorphism, animacy, likability, and intelligence). These analyses evaluated whether agent type influenced participants’ perceptual evaluations. The overall multivariate effect of agent condition was significant, Wilks’ Λ = .71, *F*(8, 200) = 4.75, *p* < .001, partial η^2^ = .16, indicating that perceptions of the agents varied across conditions (Fig. 6). Assumptions of equality of covariance matrices (Box’s *M* = 18.06, *p* = .65) and homogeneity of variances (all Levene’s *p* > .05) were satisfied.

Follow-up univariate ANOVAs showed a significant effect of agent condition on perceived anthropomorphism, *F*(2, 103) = 14.86, *p* < .001, partial η^2^ = .22. Bonferroni-adjusted post hoc comparisons indicated that the Human Hand (*M* = 3.08, *SD* = 0.73) was rated as significantly more anthropomorphic than the Humanoid Hand (*M* = 2.13, *SD* = 0.83, *p* < .001). There was no significant difference between the Humanoid Hand and the Tripod Gripper Hand (*M* = 2.13, *SD* = 0.95, both *p*s > .05). No other subscales showed significant differences by agent condition: animacy, *F*(2, 103) = 2.34, *p >* .05; likability, *F*(2, 103) = 2.74, *p >* .05; and intelligence, *F*(2, 103) = 2.28, *p >* .05. Descriptively, the Human Hand was rated higher on animacy (*M* = 2.99, *SD* = 0.79) and intelligence (*M* = 3.31, *SD* = 0.82), while the Humanoid Hand was rated highest on likability (*M* = 3.11, *SD* = 0.85), but these differences were not statistically significant (all *p*s > .05). These findings demonstrate that the perceptions of the agents were differentiated primarily due to the extent participants were attributing human-like qualities to the agents.

#### 3.2.2. Mediation model of agent condition, perceived traits, and trust

To address RQ4, mediation analyses were conducted to examine whether differences in trust between agent conditions were explained by perceived agent attributes. A parallel mediation model (PROCESS Model 4; Hayes, 2017) with 5000 bootstrap samples and 95% confidence intervals was conducted to examine whether the perceptions of anthropomorphism, animacy, likability, and intelligence mediated the relationship between agent condition and trust. The categorical predictor (agent condition) was dummy coded with the Human Hand as the reference group. Assumption checks indicated that multicollinearity was acceptable (all VIFs <2.47) and heteroskedasticity was not detected (Breusch-Pagan *p* > .05).

The overall model to predict trust when all mediators and agent conditions were entered simultaneously into the model was significant, *F*(6, 99) = 15.66, *p* < .001, *R*^2^ = .49, adj. *R*^2^ = .46, explaining approximately 49% of the variance in trust ratings (see Fig. 7). Each mediator was regressed on the agent condition to examine how perceptions of anthropomorphism, animacy, likability, and intelligence varied across agents (model statistics reported in Table 10). Agent condition significantly predicted anthropomorphism *F*(2, 103) = 17.87, *p* < .001, *R*^2^ =.26, but not animacy, *F*(2, 103) = 2.20, *p* > .05, *R*^2^ = .04, likability, *F*(2, 103) = 2.28, *p* > .05, *R*^2^ = .04, or intelligence, *F*(2, 103) = 2.31, *p* > .05, *R*^2^ = .04. Relative to the Human Hand, both the Humanoid Hand and Tripod Gripper Hands were perceived as significantly less anthropomorphic. While the Humanoid Hand did not significantly differ from the Human Hand in perceived intelligence, the Tripod Gripper Hand was rated as significantly less intelligent than the Human Hand. Neither robot differed significantly from the Human Hand in animacy or likability (all *p*s > .05). These results suggest that both robots were perceived as less human-like than the Human Hand, and the Tripod Gripper Hand was viewed as less intelligent than the Human Hand. There were no significant differences in the likability or animacy of the robots compared to the human.

In the total-effect model which examined the overall effect of the dummy-coded agent condition on trust without accounting for the influence of the mediators, participants reported significant differences in trust based on agent condition *F*(2, 103) = 3.37, *p* < .05, *R*^2^ = .06, adj. *R*^2^ = .04. The total effects suggest that, before accounting for perceived traits, the Humanoid Hand elicited greater trust than the Human Hand, while the Tripod Gripper Hand did not differ in trust compared to the Human Hand (Table 10). Overall, the direct effects indicate that after accounting for the influence of anthropomorphism, animacy, likability, and intelligence, the relationship between agent condition and trust was no longer statistically significant *F*(2, 99) = 1.68, *p* > .05, *R*^2^ change = .02. This suggests that differences in trust between agents were largely explained by participants’ perceptions of the agents’ attributes.

Across conditions, perceived likability and perceived intelligence emerged as significant positive predictors of trust. Anthropomorphism and animacy (*p*s > .05) were not significant predictors once the other variables were included (Table 10). These findings indicate that across the agents, participants’ trust was primarily shaped by how likable and intelligent they perceived the agent to be, rather than its perceived anthropomorphism or animacy.

Bootstrapped analyses with 5000 bootstrap samples revealed a significant indirect pathway specific to the Tripod Gripper Hand condition (Table 11). The Tripod Gripped Hand condition showed a significant negative association with trust through perceptions of intelligence. No other indirect effects were significant (Table 11, all other 95% confidence intervals included zero). Although the total effect of the Tripod Gripper Hand condition on trust was nonsignificant, the indirect path through perceived intelligence was significant, this pattern provides evidence of a significant indirect pathway from agent condition to trust through perceived intelligence. This indicates that the effect of the Tripod Gripper Hand on trust ratings operated through the mediator of perceived intelligence rather than through a direct effect of agent condition. These findings suggest that exposure to the Tripod Gripper Hand may have lowered trust indirectly by reducing perceived intelligence, yet the nonsignificant total effect could also indicate that the overall relationship between agent condition and trust was not clearly detectable for the Tripod Gripper Hand in the present sample (Zhao et al., 2010). For the Humanoid Hand, none of the indirect pathways were statistically significant, suggesting that trust in the Humanoid Hand did not statistically differ from that of the Human Hand once trait perceptions were accounted for. Overall, these findings indicate that trust in the agents was primarily shaped by perceived likability and intelligence, and that the lower trust associated with the Tripod Gripper Hand emerged indirectly through reduced perceptions of intelligence.

## 4. Discussion

The present study examined how pointing gestures are interpreted and utilized when produced by a human agent or by robotic agents with different appearances. Eye tracking measures of anticipatory visual attention and subjective evaluations of trust and perceived agent attributes were used to examine the communicative use of nonverbal cues, the relationship between reliance and trust, and the role of anthropomorphism, animacy, likability, and perceived intelligence in mediating the relationship between agent condition and trust ratings. The following sections discuss these findings in relation to prior work on nonverbal communication, trust as a framing mechanism for information use, and how expectations about agent identity and attributes influence interactions.

### 4.1. Utilization of pointing cues

A central aim of this study was to examine whether pointing gestures were utilized as communicative cues when the gestures were produced by human or robot agents. Patterns of visual attention during the Prediction phase were used as the primary indicator of cue utilization. Anticipatory looking reflects participants’ use of ostensive cues to form expectations about upcoming events (Csibra & Gergely, 2009; Joyce et al., 2015). During the Prediction phase of the study, participants in the Human Hand and Humanoid Hand conditions, but not the Tripod Gripper Hand condition, allocated greater anticipatory attention to the cued target location. This pattern indicated that in the Human Hand and Humanoid Hand conditions, participants utilized information provided by the agents to predict the location of the toy’s appearance, even under conditions of imperfect cue reliability (i.e., there was an equal chance that the trial would have been incongruent or congruent for cued trials), whereas the pointing produced by the Tripod Gripper Hand was not treated as informative and did not elicit anticipatory expectations. As noted in the design section, the Tripod Gripper Hand produced a whole-gripper point rather than an index-finger point. Therefore, reduced cue utilization in the Tripod Gripper Hand condition may reflect both agent morphology and the visual form of the cue produced by that morphology.

During the other phases of the experiment, attention did not meaningfully differ across agent conditions. Attention during the Action phase reflected overall allocation to the agent while it was visible on the screen. Attention during the Event phase reflected reactions to the reveal of the feathered toy. Since participants showed comparable meaningful patterns of visual attention across agent conditions during both the Action and Event phases, the differences observed during the Prediction phase are particularly informative. Specifically, participants attended to the agents and the toy’s appearance in a similar manner across conditions yet engaged in anticipatory looking toward the cued location only when interacting with the Human Hand and Humanoid Hand. This has provided direct behavioral evidence that individuals interpret pointing as communicative from other humans and from a humanoid robot and utilized the provided information to guide their behavior, but the same effect was not seen from participants viewing the non-humanoid robot, despite consistency in pointing and cue reliability across all agents. This pattern is important to recognize, as it demonstrates that a robot simply producing a gesture is not sufficient for it to function as an effective nonverbal communicator. When designing social robots that rely in some way on producing nonverbal communication, ensuring the gestures are perceived as socially meaningful is essential for the human to use the information communicated.

### 4.2. Intersection of trust and reliance

A second aim of this study was to examine how subjective trust related to participant utilization of nonverbal pointing cues. Prior work has relied on self-report measures of trust to infer reliance despite limited evidence linking trust judgments to behavior during the task (Law & Scheutz, 2020; Lee & See, 2004; Robinette et al., 2017). In the present study, trust was assessed following task completion, allowing subjective evaluations of the agent to be examined alongside anticipatory visual attention collected during the interaction, to explore how trust and reliance on cues functioned. Our findings reinforced the distinction between self-reported trust and actual reliance on provided information during a task, as trust scores were not found to be related to cue utilization. These findings caution against interpreting trust in robots as a direct indicator of potential reliance and highlight the value of integrating both objective and subjective measures when examining trust and utilization of robotic information. This distinction is important because if trust judgements do not directly reflect the utilization of information from robots in HRIs than simply improving trust perceptions of robots will not be sufficient to ensure humans actually rely on robotic guidance.

### 4.3. Perceived agent attributes and trust

The final aim of this study was to examine how perceived agent attributes shaped trust and whether differences in trust across agent types could be explained by the perceived attributes. Prior work has emphasized that trust in robots largely reflects evaluative judgements of their perceived characteristics (Christoforakos et al., 2021; Hancock et al., 2011; McKenna et al., 2024). Building on this perspective, the present study directly compared robotic and human agents that differed in human-likeness and hand dexterity while producing pointing gestures, allowing perceived attributes and trust to be examined within a controlled context. This allowed for the direct comparison of human and robot differences in trust and the identification of which dimensions were most impactful on trust.

Perceived anthropomorphism was the only attribute that differed across agent conditions, with the Human Hand rated as more anthropomorphic than either robotic agent. However, anthropomorphism did not predict trust once other perceived attributes were considered. Recent literature is strengthening the finding that trust is often context-dependent and may be attenuated when broader evaluative traits and task types are considered (Christoforakos et al., 2021; Onnasch & Hildebrandt, 2021; Roesler et al., 2024). Although perceived anthropomorphism can shape expectations about communicative intent (Kiesler et al., 2008; Złotowski et al., 2016), the present results suggest that perceived human-likeness alone is insufficient to explain differing trust judgments in the context of nonverbal gestural communication.

Perceived animacy did not significantly predict trust in the present study. Although animacy is discussed as an important factor in human-robot interactions (Bartneck et al., 2008), its relationship with trust has been less consistently demonstrated than that of other evaluative attributes. Conceptual overlap between animacy and anthropomorphism has also been noted in prior work (Bartneck et al., 2008; Weiss & Bartneck, 2015), which may limit the extent to which animacy uniquely contributes to trust judgments when multiple perceived traits are considered simultaneously. In the present study, animacy did not explain additional variance in trust beyond other perceived attributes, indicating that perceptions of animacy are not sufficient to account for trust judgments in this nonverbal gestural context.

Perceived intelligence and likability emerged as the strongest predictors of trust in the agent conditions, highlighting the importance of evaluative social traits in trust formation during nonverbal communication. Higher perceived intelligence and higher perceived likability were each associated with greater trust, despite these trait ratings not significantly differing across agent conditions at the group level. This pattern aligns with prior work positioning factors related to perceived intelligence, such as capability and reliability, as a robust predictor of trust in automated and robotic systems (Hancock et al., 2011; Schaefer et al., 2012), as well as research highlighting the role of affective evaluations such as warmth and likability in supporting trust (Salem et al., 2013, 2015). The mediation analysis clarified how these evaluative perceptions related to agent differences in trust when the Human Hand served as the reference group. Compared to the Human Hand condition, the Tripod Gripper Hand was associated with lower perceived intelligence, which was in turn associated with lower trust. In contrast, no significant indirect effects were observed for the Humanoid Hand relative to the Human Hand. These findings indicate that perceived intelligence and likability were central predictors of trust overall, while perceived intelligence may have explained the observed trust difference for the Tripod Gripper Hand relative to the Human Hand.

Although differences between the agents did not translate into differences in trust through anthropomorphism or animacy in this nonverbal communication context, evaluative judgments related to perceived competence played a meaningful role in shaping trust. This pattern suggests that, even when agents perform communicative behaviors, trust may be differentially calibrated based on how capable the agent is perceived to be, rather than on human-likeness alone, especially for non-humanoid appearing robots. This finding supports the development of robots that convey appropriate human-likeness and competence when interacting with a human.

Overall, the present findings offer practical implications for the design of robots meant to utilize nonverbal cues to convey information. The pattern of cue utilization observed in this experiment is consistent with the expectations of natural pedagogy and CASA frameworks, suggesting that the interpretation of ostensive cues such as pointing may depend on the mental models individuals apply to the agent producing them, rather than on just the gesture alone.

Participants utilized pointing cues to guide their behavior from the Human Hand and the Humanoid Hand, but not the Tripod Gripper Hand, suggesting that the communicative effectiveness of a gesture may be shaped by the extent to which a non-human agent is perceived as being capable of communicative intent. When designing social robots that rely on nonverbal communication, it is essential to ensure the gestures made are perceived as socially meaningful for humans to use the information. In contexts where robots are meant to function as communicative teammates, such as in educational settings, collaborative workspaces, healthcare environments, or service positions, designers should ensure that those robots are producing gestures that are perceived as intentional and informative. Based on this study, creating robots that are perceived as highly likable and intelligent should increase trust in social robots. The present findings lay the foundation for understanding how and why information is utilized from human and robotic agents and suggests that perceived robot traits may play a central role in supporting trust in these contexts, rather than relying on just the agent’s appearance.

### 4.4. Limitations and future directions

Several limitations of the present study should be noted and provide directions for future research. First, the generalizability of the present findings should be considered in light of the diversity and complexity of real-world robotic systems and the practical design choices that were made for this study. Robots vary widely in appearance, embodiment, and communicative capabilities, and there is currently little standardization in how robots are selected across human-robot interaction studies, making cross-study comparison difficult. The present study intentionally focused on robotic arms and hands to isolate nonverbal gestural communication and to prevent participants from relying on gaze or facial cues as additional sources of information. While this design choice supported experimental control, it also limits the extent to which the findings can be directly generalized to robots equipped with faces, expressive eyes, or full-body embodiments which elicit more social attributions. As robots continue to evolve and possess rich social features, future research should examine how pointing gestures interact with other factors, such as gaze, facial expression, body posture, and in-person interaction to shape attention, trust, and reliance. The agents in this study were limited to only using pointing gestures, and the Tripod Gripper Hand specifically produced a whole-gripper point rather than an index-finger point, reflecting the lower degrees of freedom and three-digit pincer configuration of the hand. Although this cue still provided referential information toward the target location, future work could further examine how different robotic pointing forms influence cue utilization across agent types.

In the same vein, the use of green gloves to cover the hands and arms of the presented agents does limit the ecological scope of the findings. The use of gloves reduced the perceptual differences across the agents and allowed for the meaningful detection of cue utilization by reducing the effect of novelty or possible preferences for the robots’ more interesting appearances compared to a human agent. However, it is entirely possible that the uncovered agents would have elicited different results. The findings should be interpreted as evidence about cue utilization in response to visually controlled pointing gestures from different human or robot agent types. Future work should directly compare covered and uncovered versions of human and robotic hands to determine how naturalistic appearance influences attention to the agent and use of the pointing cue. Due to these constraints and the lack of an explicit manipulation check from participants, future studies should make efforts to include direct agent-identification items to assess how participants categorized the hand they viewed and how those categorizations may relate to cue utilization, perceived agent attributes, and trust.

Additionally, the agents in this experiment were not co-located in the room with the participant. All interaction with the agent viewed was presented through pre-recorded videos over a computer monitor. While all three agent presentations were kept consistent to the greatest extent possible (in terms of appearance, size, movement, etc.) to facilitate accurate eyetracking data collection, nonverbal communication interpretation and agent perception between humans and robots that are co-located may differ from the findings presented here. Future studies should aim to conduct research observing how these findings may differ when the actual humans or robot agents are presented in their physical forms. A related consideration concerns participants’ perceptions of agent autonomy. In the present study, all agents’ movements were pre-programmed, but participants were not informed of this, and individual assumptions about whether the pointing gesture was self-initiated or operator-controlled may have varied. Prior work suggests that perceived autonomy can influence trust in robotic agents (Hancock et al., 2011), and it is plausible that perceptions of agency moderated how participants interpreted and responded to the pointing gestures observed here. Examining whether trust and cue utilization differ as a function of explicitly manipulated or measured perceived agency represents an important direction for future research in nonverbal HRI contexts.

Second, the frequency of correct and incorrect pointing cues was held constant, and reliability was not manipulated as an experimental factor. This design choice was intentional, as the primary goal was to examine whether nonverbal gestures were treated as communicative and how perceived agent attributes related to trust under controlled conditions of imperfect reliability. Holding the reliability rate constant allowed differences in cue utilization and trust to be attributed to agent type and perceived traits rather than to learning effects driven by changing performance. However, trust in real-world human-robot interaction often develops dynamically as agents demonstrate reliability, make errors, or adapt over time. Future research should systematically vary reliability to examine how trust and the interpretation of nonverbal cues evolve across repeated interactions. This study also examined trust only as a post-interaction evaluation rather than as a dynamic construct evolving over time. While this approach allowed trust judgments to be examined alongside behavioral measures collected during the interaction, it does not capture how trust may fluctuate as agents demonstrate reliability, inconsistency, or adaptation across repeated interactions. This is a limitation of the study and should be acknowledged when interpreting the results of the experiment. It is likely that the relationship between cue utilization and trust would vary based on measurement times. Future work should consider longitudinal designs or repeated measures approaches that track changes in trust and cue utilization over time, particularly in contexts where robots are expected to serve as ongoing communicative partners, and as reliability of the agents change over time. Trust calibration is increasingly understood as a dynamic process (Lee & See, 2004), and the design of the current study limits the extent to which findings can reflect how individuals adjust their reliance on nonverbal cues over time.

Third, the participant sample exhibited a gender imbalance, with a greater proportion of female participants, reflecting the demographics of the recruitment population. Although gender was not a primary variable of interest in the present study, prior work suggests that individual differences such as gender may influence perceptions of robots and attitudes toward technology (Kislev, 2023; Russo et al., 2025). A major limitation of this study is the gender imbalance of the participants recruited. The majority female sample limits the generalizability of these findings across different genders and may have influenced the findings of this experiment. It is possible that non-female individuals would perceive and attend to robotic arms in different ways that this study was not adequately powered to detect. As such, future studies should aim to recruit more balanced gender samples to examine whether individual differences change the interpretation of nonverbal cues, perceived agent attributes, or trust formation in human-robot interaction.

Overall, these limitations point to clear priorities for future work. Future studies should build directly on this foundational nonverbal communication study by A) integrating pointing with other social cues (e.g., other gestures, gaze, facial expressions, verbal communication, tactile exchanges, etc.), B) manipulating reliability and error types (e.g., slips, lapses, mistakes, and violations) across different robotic agents to observe changes in trust, and C) recruiting more diverse and gender-balanced participant samples to explicitly test how individual differences influence nonverbal information use from non-human agents. Addressing these challenges will be essential for advancing both theory and practice in HRI as robots are increasingly deployed as communicative partners in applied settings.

## 5. Conclusion

Effective human robot communication depends not only on the presence of nonverbal cues, but on whether those cues are interpreted as intentional and informative (Saunderson & Nejat, 2019; Sauppé & Mutlu, 2014). The results indicate that pointing gestures were utilized only when produced by agents that were treated as communicative. The findings also suggest that human-likeness should not be treated as the primary driver of trust. Although anthropomorphism was perceived differently across the agents, it did not uniquely account for trust once other evaluative traits were considered. This pattern reinforces the limitations of design approaches that prioritize increasing human-like form as a means of fostering trust. In nonverbal communicative tasks centered on information exchange, trust appears to depend less on how human-like an agent looks and more on how it is evaluated as a communicator. Evaluative perceptions related to perceived intelligence and likability played a more central role in shaping agent trust. Perceived intelligence, in particular, accounted for trust differences between the Tripod Gripper Hand and the Human Hand, indicating that subjective judgments of competence and capability were especially influential for robots that were non-human like. Likability also contributed to trust across agents, underscoring the importance of affective impressions alongside competence-based evaluations.

More broadly, the results emphasize the importance of aligning robot design with the social expectations associated with the robot’s role. When robots are positioned as sources of information, trust can be shaped by whether they are perceived as likable and intelligent rather than by their resemblance to humans. Designing robots that convey competence and communicative relevance may better support trust calibration and information use in human-robot interaction.

## Supplementary Material

1

2

3

4

5

6

7

8

9

10

11

12

13

14

15

16

17

18

19

## Figures and Tables

**Fig. 1.**
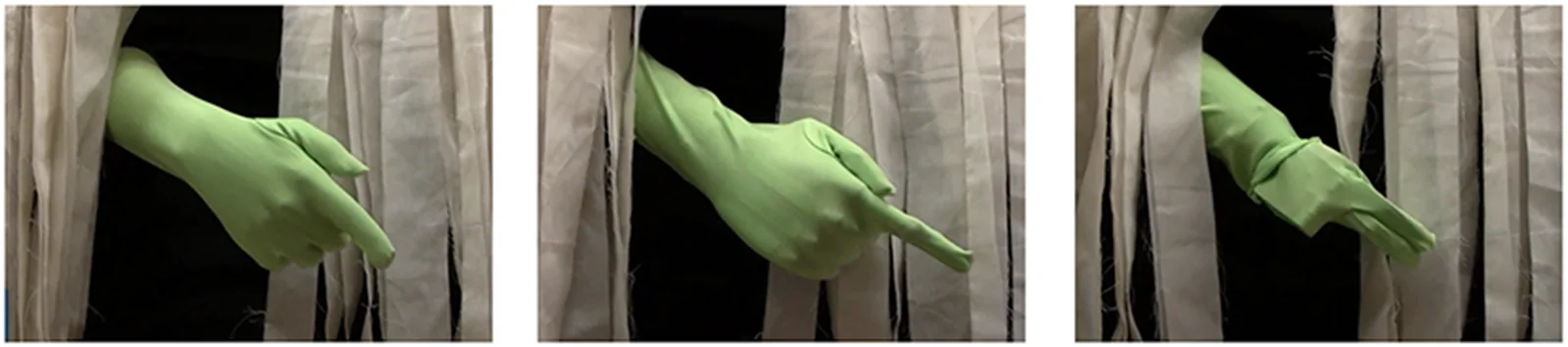
Still Images of the Agent Stimuli *Note*. The Human Hand, Humanoid Hand, and Tripod Gripper Hand (left to right) used in the experiment. These still images were captured from the videos presented to the participants during the Action phase of the experiment.

**Fig. 2.**
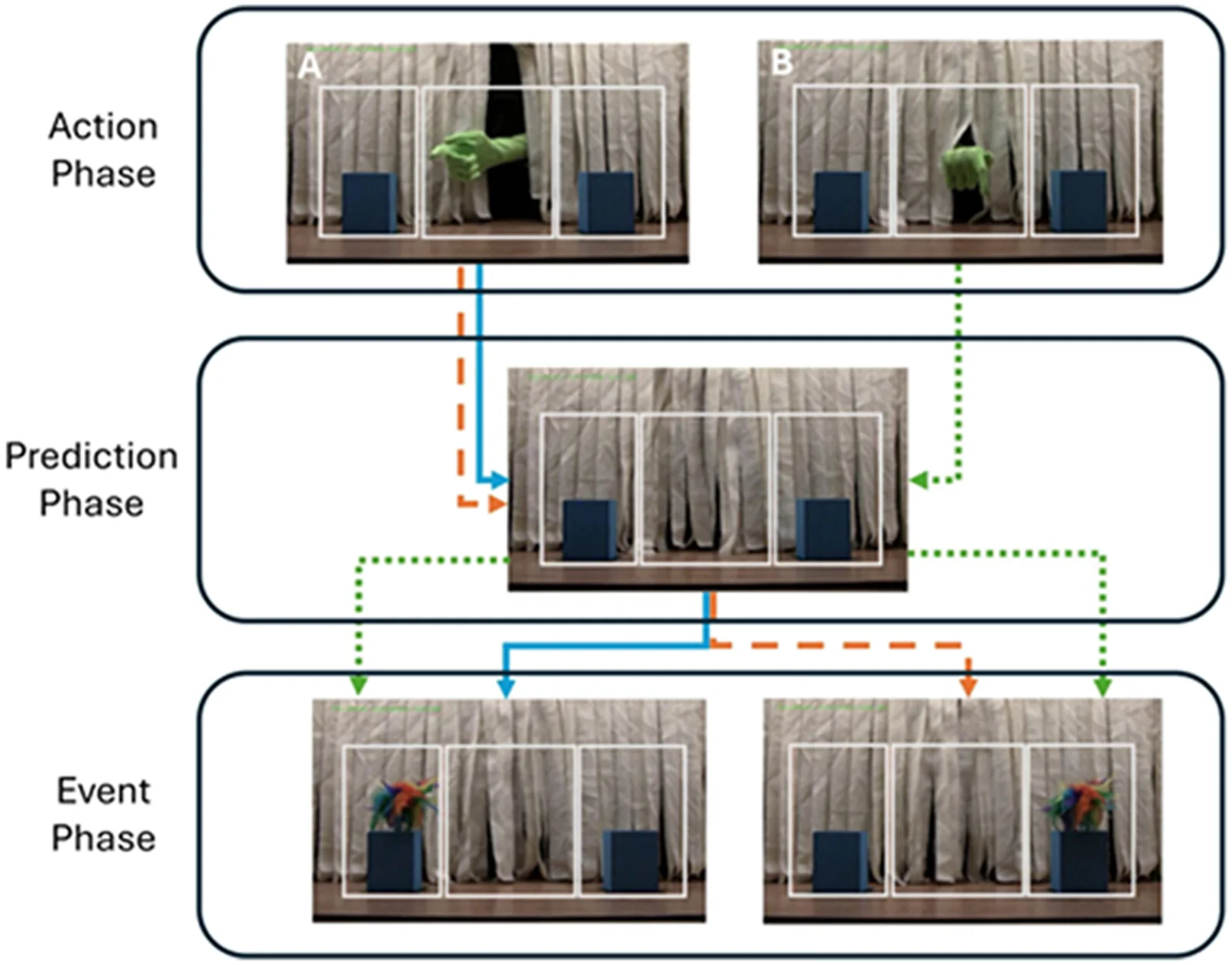
Video Trial Structure *Note*. Stills depicting the flow of the presented video trials with the Humanoid Hand agent depicted. Trial flows were the same for all agent types. Trials began with the Action phase (15 s), either a cued (panel A) or control (panel B) point. Trials then had a Prediction phase (3 s) followed by an Event phase (2 s). Congruent trial flow is depicted with solid blue arrows. Incongruent trial flow is depicted with dashed orange arrows, and control trial flows are depicted with dotted green arrows.

**Fig. 3.**
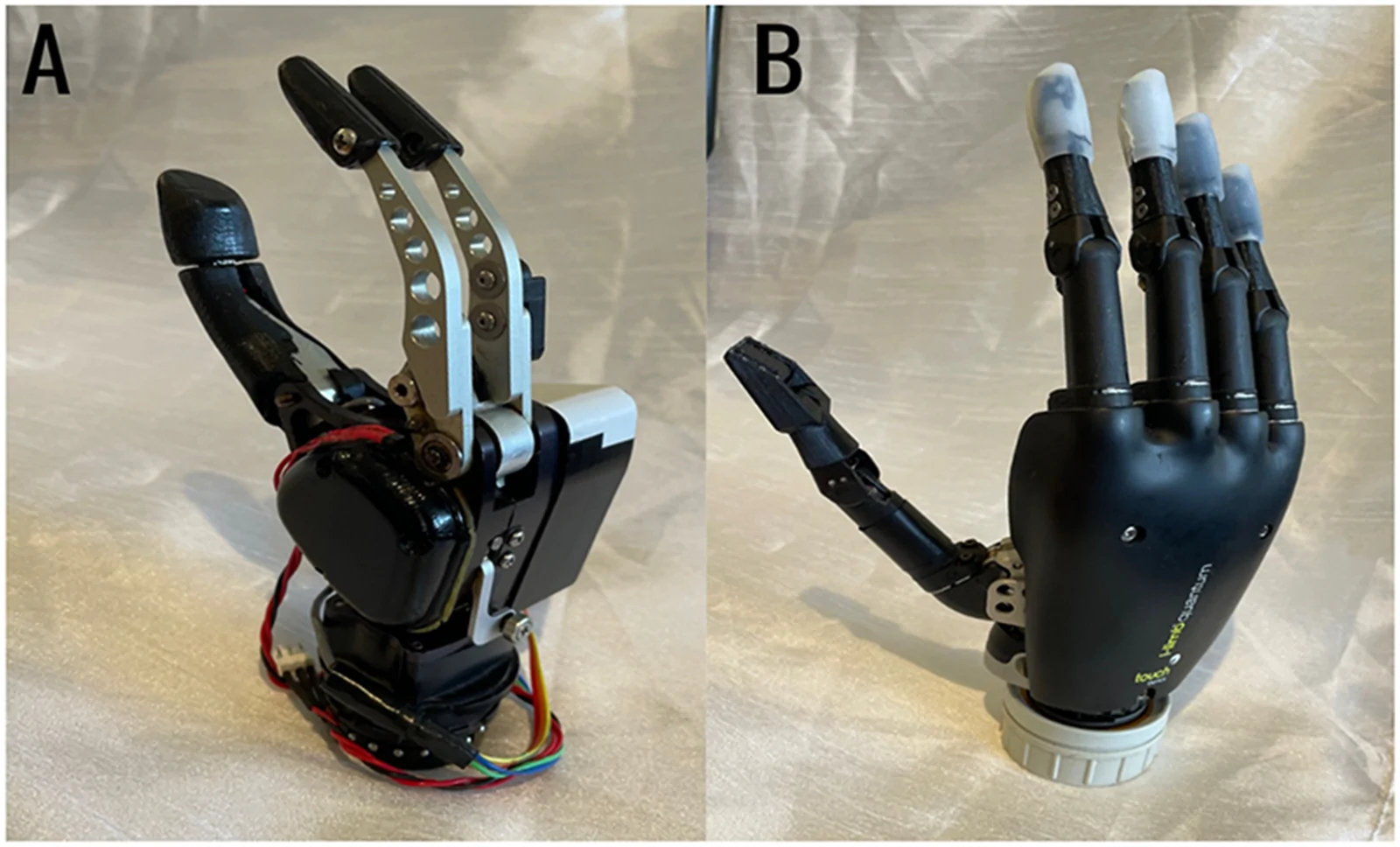
Robotic Hands Used Note. The image depicts the robot hands used in the experiment before they were covered with the green gloves. Panel A depicts the Tripod Gripper Hand. Panel B depicts the Humanoid Hand.

**Fig. 4.**
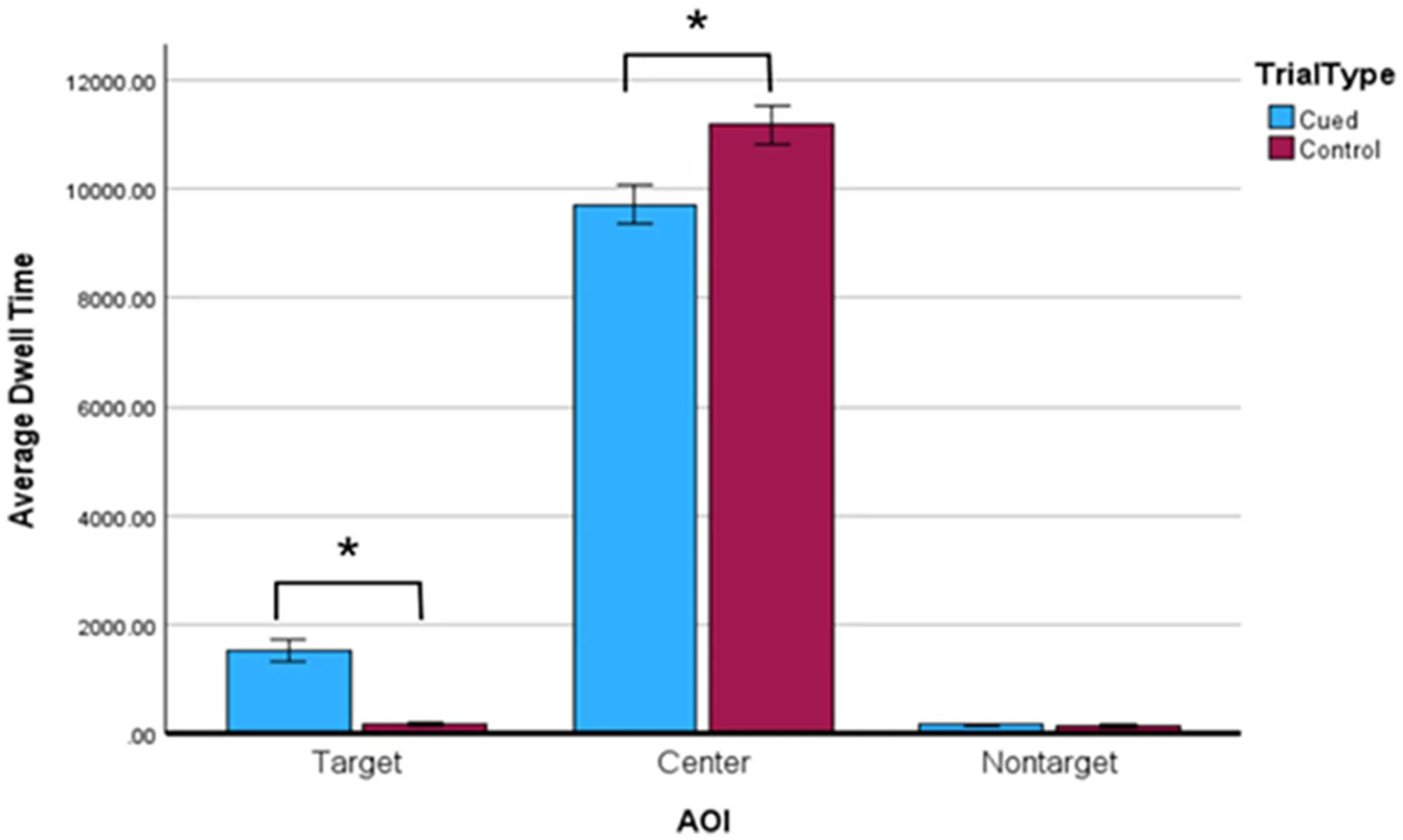
Dwell Time in Action Phase by AOI and Trial Type *Note*. Bar chart representing average dwell time in milliseconds during the Action Phase by Area of Interest and by Trial Type. Asterisks denote a statistically significant result at *p* < .05.

**Fig. 5.**
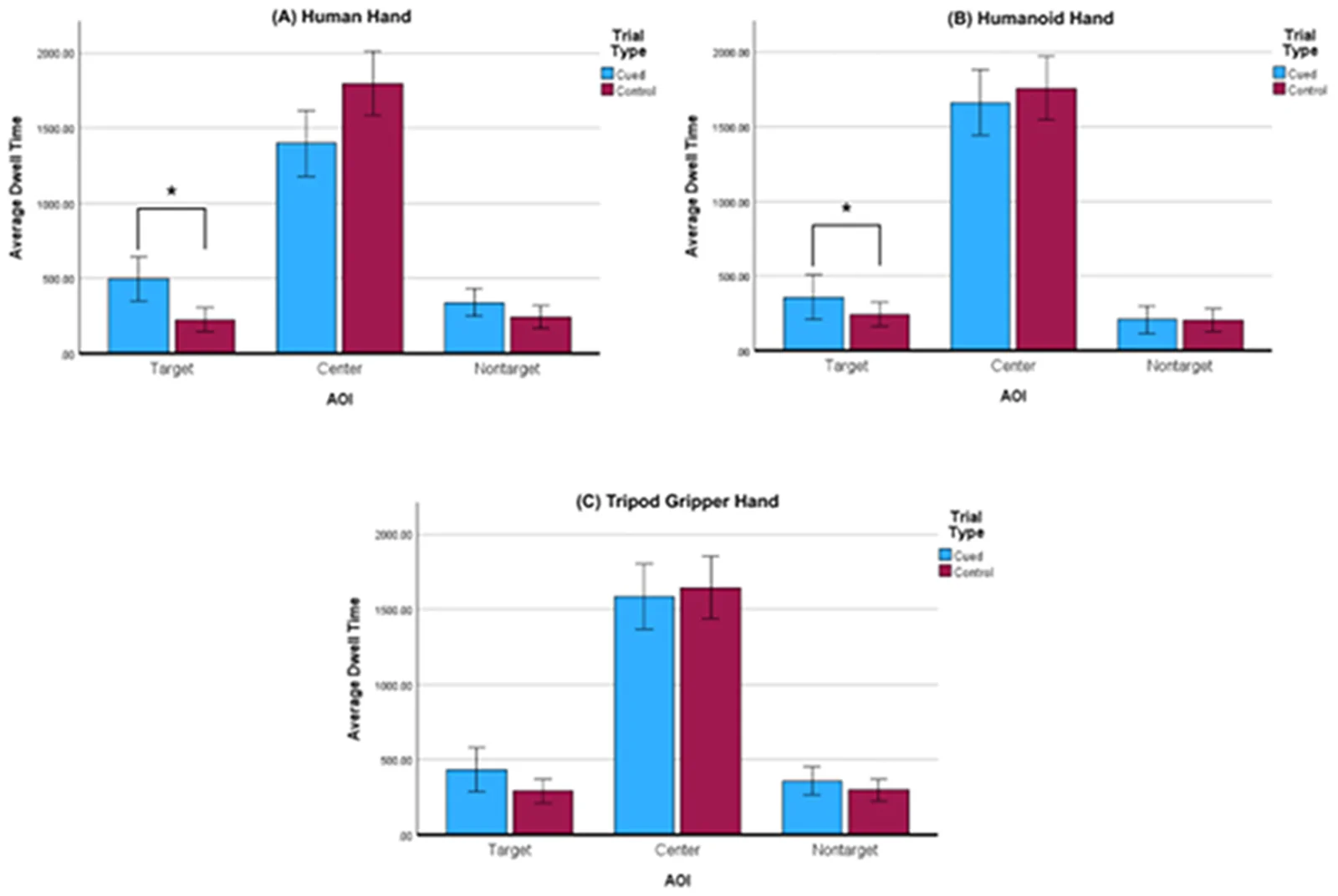
Dwell Time in Prediction Phase to AOIs by Trial Type Across Agents *Note*. Dwell time in the Prediction phase to AOIs by trial type across agent condition. Bars represent average dwell time (milliseconds) during the Prediction phase for cued and control trials across AOIs (Target, Center, Nontarget), plotted separately for (A) Human Hand, (B) Humanoid Hand, and (C) Tripod Gripper Hand. Error bars indicate 95% confidence intervals. Asterisks denote significant differences between cued and control trials within a given AOI (p < .05).

**Fig. 6.**
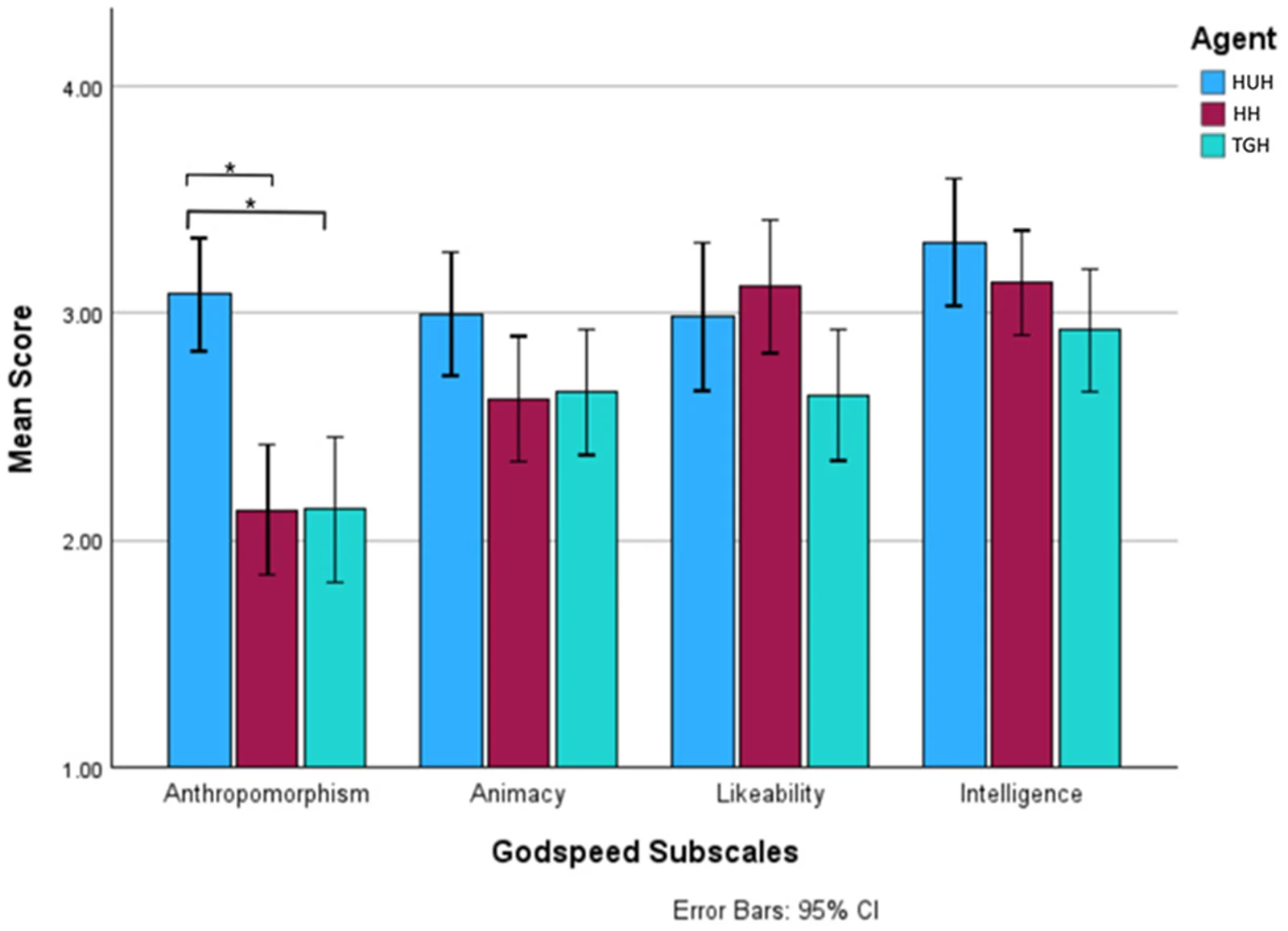
Mean Scores for Godspeed Subscale Traits Based on Agent Condition *Note*. Godspeed subscale composite means ranged from 1 to 5, with a higher score indicating a greater perception of that trait. Asterisks denote a statistically significant result at *p* < .05. HUH = Human Hand; HH = Humanoid Hand; TGH = Tripod Gripper Hand.

**Fig. 7.**
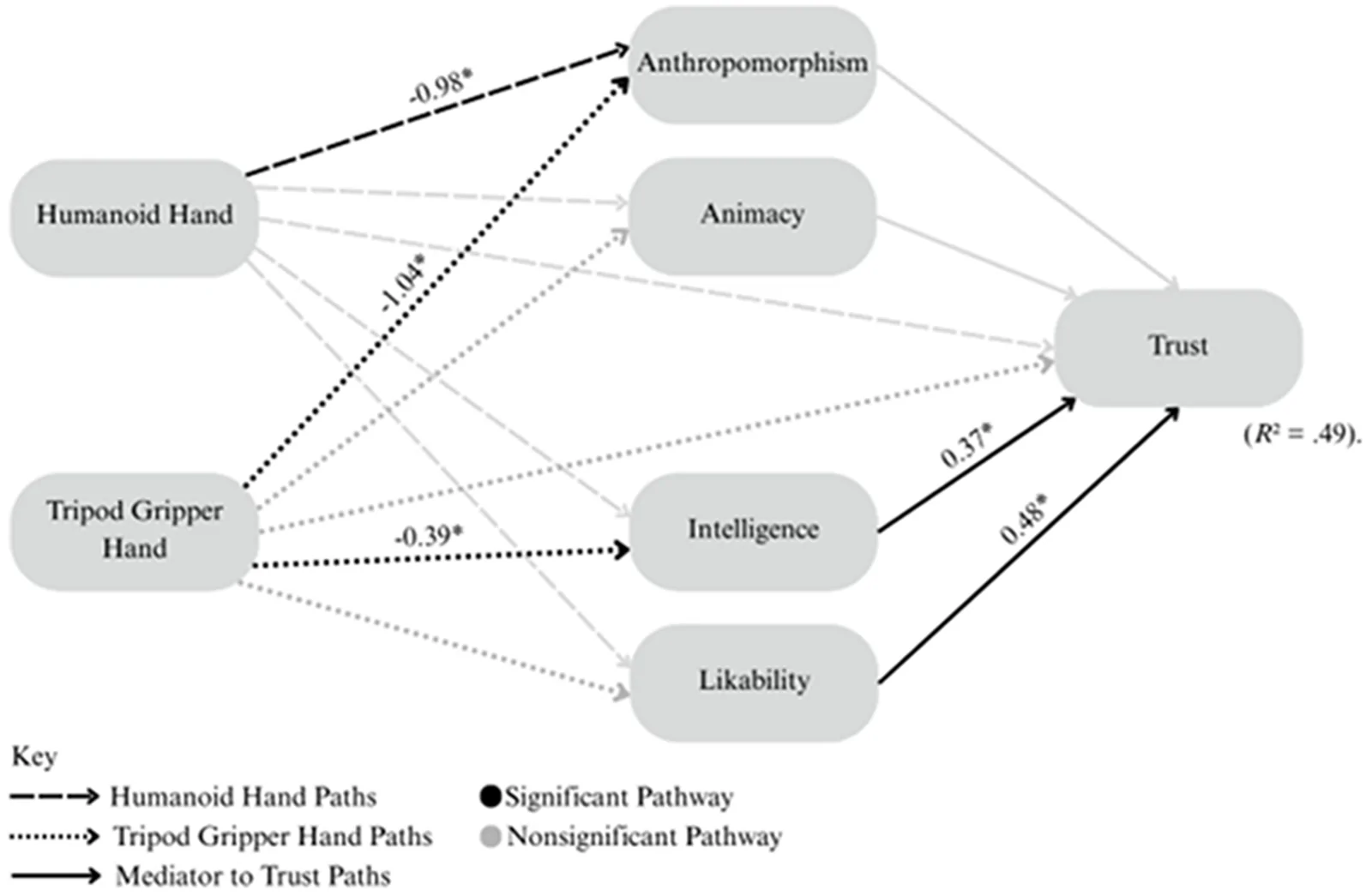
Parallel Mediation Model Testing Perceived Mediators of the Relationship Between Agent Condition and Trust *Note*. Significant unstandardized regression coefficients are displayed. The Human Hand served as the reference group for the other agents. Dashed paths represent contrasts between the Humanoid Hand and the Human Hand; dotted paths represent contrasts between the Tripod Gripper Hand and the Human Hand. Solid arrows from mediators to trust represent pooled b-paths estimated while controlling for agent condition and the other mediators. Bold black paths indicate statistically significant effects (p < .05); grey paths indicate nonsignificant effects (p ≥ .05). The only significant indirect effect was observed for the Tripod Gripper Hand through perceived intelligence into trust. The model explained approximately 49% of the variance in trust scores.

**Table 1:** Effects of perceived robot attributes on trust and nonverbal communication.

Dimension	Definition	General Effect on Trust	Role in Nonverbal Robotic Communication
Anthropomorphism	The degree to which a robot is perceived as having human-like traits such as form, behavior, or characteristics.	Greater perceived human-likeness often increases trust, although effects vary by context (Duffy, 2003; Hancock et al., 2011; Roesler et al., 2021).	Greater human-like physical form can elicit human expectations for communicative behavior, increasing the likelihood that cues are considered informative (Kiesler et al., 2008; Złotowski et al., 2016).
Animacy	The extent of aliveness, human-likeness, or naturalness of movement attributed to a robot.	Animacy has been less examined in direct relation to trust, but evidence suggests that more natural and intelligible robot movement can support trust, particularly when robot behavior aligns with user expectations (Hancock et al., 2011; Hostettler et al., 2023).	Natural, human-like movement can increase the likelihood that nonverbal cues are interpreted as intentional and meaningful, whereas mismatches between a robot’s appearance and its movement patterns may reduce trust and robot credibility (Hostettler et al., 2023; Złotowski et al., 2016).
Intelligence	The extent to which an individual attributes capabilities, skills, and competence to a robot.	Higher perceived intelligence is associated with greater trust, often independent of a robot’s performance alone (Hancock et al., 2011; Schaefer et al., 2012).	When a robot is perceived as intelligent, its behavior is more likely to be interpreted as purposeful and reliable, which can increase users’ willingness to rely on its nonverbal cues (Salem et al., 2015; Schaefer et al., 2012).
Likability	The extent to which an individual attributes friendliness and social appeal to a robot.	Greater perceived robot likability is associated with higher trust ratings and can mitigate declines in trust following errors (Hancock et al., 2011; Salem et al., 2015)	Higher perceived likability can shape affective responses to a robot, increasing engagement and positive reception of its nonverbal behaviors (Hancock et al., 2011; Salem et al., 2013).

*Note*. This table serves as a summary of high impact literature to summarize the general trends in the current literature surrounding robotics perception dimensions, and their relation to trust and nonverbal communication, but is in no way meant to be an exhaustive review. These definitions were influenced by Bartneck et al., 2008.

**Table 2:** Descriptive statistics of the samples sex and race.

Condition									
		HUH		HH		TGH		Total	
		N	%	N	%	N	%	N	%
Sex	Female	28	80	28	80	23	63.9	79	74.5
	Male	7	20	7	20	13	36.1	27	25.5
	Total	35	100	35	100	36	100	106	100
Race	African American	5	14.3	1	2.9	4	11.1	10	9.4
	Asian	1	2.9	1	2.9	0	0.0	2	1.9
	Hispanic	6	17.1	9	25.7	7	19.4	22	20.8
	White	17	48.6	20	57.1	15	41.7	52	49.1
	Multiracial	5	14.3	4	11.4	10	27.8	19	17.9
	Other	1	0.9	0	0.0	0	0.0	1	0.9
	Total	35	100	35	100	36	100	106	100

*Note*. Descriptive statistics table for participant sex and race in this sample. The sample consisted of 106 participants and was mostly female and White.

**Table 3:** Average Dwell Time During the Action phase.

Trial Type	AOI	Agent Condition	Block 1 Mean (SD)	Block 2 Mean (SD)	Block 3 Mean (SD)
Cue Trial	Center	HUH	9822.71 (1929.92)	9645.25 (2079.59)	9663.23 (1779.72)
		HH	9368.79 (1789.86)	9273.76 (1986.86)	8955.93 (2049.16)
		TGH	10479.53 (1692.64)	10378.80 (1804.79)	9892.07 (1963.35)
	Nontarget	HUH	147.03 (146.28)	114.55 (138.23)	157.95 (153.69)
		HH	123.85 (109.04)	109.13 (138.23)	117.23 (90.98)
		TGH	225.72 (170.14)	190.81 (188.61)	223.06 (165.38)
	Target	HUH	1468.69 (1172.66)	1485.42 (1381.38)	1487.52 (1222.76)
		HH	1943.14 (1088.62)	1811.74 (1271.33)	1875.50 (1059.73)
		TGH	1154.74 (883.69)	1261.41 (1089.10)	1194.60 (938.98)
Control Trial	Center	HUH	11484.01 (1757.80)	11275.15 (2257.26)	11219.32 (1838.07)
		HH	11116.71 (1896.87)	10787.07 (2473.50)	10519.09 (2222.99)
		TGH	11607.47 (1446.67)	11556.57 (1657.82)	11060.75 (1928.61)
	Nontarget	HUH	114.77 (134.54)	120.83 (196.63)	109.84 (167.10)
		HH	120.77 (131.44)	107.71 (190.97)	118.53 (143.61)
		TGH	186.51 (166.05)	211.30 (254.84)	185.13 (203.40)
	Target	HUH	138.01 (150.87)	137.93 (190.71)	155.84 (218.18)
		HH	131.55 (148.82)	109.08 (170.14)	152.81 (198.85)
		TGH	209.13 (190.33)	228.25 (248.17)	232.14 (277.07)

*Note:* Average dwell time in milliseconds during the trial type (Cued or Control) by each Area of Interest (Center, Nontarget, and Target) for the three agent conditions (HUH, HH, TGH) during the three blocks (Block 1, Block 2, Block 3) of the Action phase of the experiment. HUH = Human Hand; HH = Humanoid Hand; TGH = Tripod Gripper Hand.

**Table 4:** Repeated measures ANOVA results for action phase.

Variables	df	F	*p*	*Partial Eta* *Squared*
Agent	2, 103	1.06	.35	.02
Block	1.29, 116.99	13.49*	<.001	.116
Block * Agent	2.58, 233.97	2.19	.102	.041
Trial Type	1, 94	1.78	.186	.017
Trial Type * Agent	2, 188	2.94	.057	.054
AOI	1.13, 106.96	2922.83*	<.001	.966
AOI * Agent	2.25, 211.83	2.44	.085	.045
Block * Trial Type	1.35, 127.16	0.42	.578	.004
Block * Trial Type * Agent	2.71, 254.63	0.42	.720	.008
Block * AOI	1.64, 154.23	15.21*	<.001	.129
Block * AOI * Agent	3.28, 308.67	1.64	.177	.031
Trial Type * AOI	1.11, 104.29	184.65*	<.001	.642
Trial Type * AOI * Agent	2.22, 208.67	3.15*	.042	.058
Block * Trial Type * AOI	1.59, 149.85	0.49	.570	.005
Block * Trial Type * AOI * Agent	3.19, 300.21	0.60	.628	.011

*Note*. Results from RM ANOVA for dwell time in milliseconds during the Action Phase with agent (Human Hand, Humanoid Hand, Tripod Gripper Hand) as the between-subjects factor and AOI (Center, Nontarget, Target), Trial Type (cued and control), and Block (Block 1, Block 2, Block 3) as the within-subject factors. Mauchly’s Test of Sphericity indicated that the assumption of sphericity was violated; therefore, the corrected results using the Greenhouse-Geisser adjustment are reported. Asterisks denote a statistically significant result at *p* < .05.

**Table 5:** Average dwell time during the prediction phase.

Trial Type	AOI	Agent Condition	Block 1 Mean (SD)	Block 2 Mean (SD)	Block 3 Mean (SD)
Cue Trial	Center	HUH	1426.37 (589.05)	1347.50 (640.44)	1419.40 (688.52)
		HH	1665.70 (613.23)	1635.93 (709.63)	1687.11 (727.70)
		TGH	1632.54 (636.57)	1608.54 (721.14)	1511.78 (725.42)
	Nontarget	HUH	313.66 (269.21)	345.06 (346.12)	356.48 (337.75)
		HH	222.03 (192.87)	211.52 (250.60)	193.66 (206.69)
		TGH	326.73 (254.06)	341.85 (340.04)	408.77 (395.32)
	Target	HUH	509.81 (458.81)	513.95 (535.14)	465.71 (528.15)
		HH	386.50 (384.41)	339.84 (458.21)	351.56 (463.41)
		TGH	429.55 (411.03)	437.98 (459.06)	438.05 (433.84)
Control Trial	Center	HUH	1820.05 (566.43)	1788.16 (700.74)	1780.17 (700.39)
		HH	1774.81 (578.23)	1739.34 (762.23)	1758.83 (673.19)
		TGH	1727.52 (613.74)	1647.98 (713.98)	1554.06 (724.31)
	Nontarget	HUH	239.11 (240.92)	214.43 (228.42)	265.84 (327.98)
		HH	210.55 (198.75)	200.10 (251.65)	204.29 (223.86)
		TGH	275.77 (221.90)	276.58 (285.97)	335.32 (315.77)
	Target	HUH	235.36 (229.44)	247.67 (307.43)	192.86 (232.34)
		HH	256.07 (223.69)	235.33 (264.01)	238.72 (218.38)
		TGH	279.82 (260.92)	298.53 (349.82)	292.69 (297.46)

*Note*. Average dwell time in milliseconds during each trial type (Cued and Control) by each Area of Interest (Nontarget, Center, and Target) for the three agent conditions (HUH = Human Hand; HH = Humanoid Hand; TGH = Tripod Gripper Hand) during the Prediction phase of the experiment.

**Table 6:** RM ANOVA results for prediction phase.

Variables	df	F	*p*	Partial eta squared
Agent	2, 103	0.43	.651	.008
Block	1.37, 128.90	2.90	.077	.027
Block * Agent	2.74, 257.58	0.46	.695	.009
Trial Type	1, 94	2.86	.094	.027
Trial Type * Agent	2, 188	3.37*	.038	.061
AOI	1.11, 104.59	276.77*	<.001	.729
AOI * Agent	2.23, 209.41	0.63	.551	.012
Block * Trial Type	1.32, 124.42	0.88	.378	.008
Block * Trial Type * Agent	2.65, 249.62	0.44	.700	.008
Block * AOI	1.89, 178.34	1.78	.173	.017
Block * AOI * Agent	3.79, 356.06	1.65	.166	.031
Trial Type * AOI	1.35, 126.87	20.58*	<.001	.167
Trial Type * AOI * Agent	2.70, 253.64	4.15*	.010	.075
Block * Trial Type * AOI	2.29, 215.39	0.43	.680	.004
Block * Trial Type * AOI * Agent	4.58, 430.85	0.23	.941	.004

*Note*. Results from RM ANOVA for time in milliseconds spent looking to the AOIs during the Prediction Phase with agent condition (Human Hand, Humanoid Hand, Tripod Gripper Hand) as the between subjects factor and AOI (Center, Nontarget, and Target), trial type (cued, control), and block (Block 1, Block 2, Block 3) as the within-subject factors. Mauchly’s Test of Sphericity indicated that the assumption of sphericity was violated; therefore, the corrected results using the Greenhouse-Geisser adjustment are reported. Asterisks denote a statistically significant result at *p* < .05.

**Table 7:** Average dwell time during the event phase.

Trial Type	AOI	Agent Condition	Block 1 Mean (SD)	Block 2 Mean (SD)	Block 3 Mean (SD)
Congruent	Center	HUH	212.23 (101.51)	188.26 (116.92)	211.31 (130.01)
		HH	210.23 (97.71)	220.57 (110.91)	195.22 (117.65)
		TGH	238.66 (96.38)	233.59 (122.73)	203.79 (125.61)
	Nontarget	HUH	46.00 (35.64)	49.67 (48.58)	38.97 (35.01)
		HH	46.80 (32.00)	42.64 (49.44)	29.93 (34.09)
		TGH	61.93 (31.33)	55.01 (48.87)	52.63 (33.37)
	Target	HUH	1352.32 (197.11)	1200.29 (356.66)	1352.82 (193.58)
		HH	1338.93 (191.55)	1201.24 (313.91)	1347.01 (206.69)
		TGH	1329.16 (176.02)	1137.25 (362.03)	1359.14 (197.48)
Incongruent	Center	HUH	235.03 (84.87)	296.85 (214.28)	220.57 (99.09)
		HH	207.73 (94.71)	282.09 (188.47)	174.26 (109.99)
		TGH	237.29 (94.38)	318.85 (178.32)	208.60 (124.53)
	Nontarget	HUH	32.90 (24.67)	31.94 (30.82)	29.96 (36.87)
		HH	34.83 (22.39)	27.81 (29.78)	40.29 (38.51)
		TGH	46.53 (22.34)	35.21 (32.63)	52.76 (38.41)
	Target	HUH	1367.35 (179.80)	1354.75 (226.16)	1370.42 (181.34)
		HH	1368.57 (188.52)	1336.79 (245.00)	1370.77 (202.47)
		TGH	1354.65 (161.40)	1376.37 (206.43)	1330.05 (193.15)
Control	Center	HUH	212.11 (91.59)	194.68 (146.69)	184.08 (120.34)
		HH	210.17 (95.62)	246.55 (159.57)	207.33 (112.12)
		TGH	231.93 (90.94)	315.54 (248.64)	219.49 (126.63)
	Nontarget	HUH	1307.37 (181.94)	1336.58 (204.65)	1314.79 (205.31)
		HH	1301.47 (178.28)	1294.77 (233.14)	1287.14 (171.39)
		TGH	1298.05 (183.68)	1311.96 (215.31)	1270.70 (219.89)
	Target	HUH	69.01 (51.58)	63.17 (57.09)	70.61 (63.10)
		HH	71.68 (50.25)	60.86 (58.16)	58.46 (66.32)
		TGH	76.37 (46.80)	69.82 (52.45)	72.56 (66.79)

*Note*. Average dwell time in milliseconds during each trial type (Congruent, Control, and Incongruent) by each Area of Interest (Center, Nontarget, and Target) across all Blocks (Block 1, Block 2, Block 3) for the three agent conditions (HUH, HH, TGH) during the Event phase of the experiment. HUH = Human Hand; HH = Humanoid Hand; TGH = Tripod Gripper Hand.

**Table 8:** RM ANOVA results for event phase.

Variables	*df*	*F*	*p*	Partial Eta Squared
Agent	2, 103	0.33	.719	.006
Block	1.43, 134.70	3.50*	.048	.033
Block * Agent	2.87, 268.59	1.07	.362	.020
Trial Type	1.73, 162.43	27.77*	<.001	.212
Trial Type * Agent	3.46, 325.01	0.67	.592	.013
AOI	1.23, 115.94	1159.75*	<.001	.918
AOI * Agent	2.47, 231.05	0.39	.724	.007
Block * Trial Type	2.05, 192.42	41.41*	<.001	.287
Block * Trial Type * Agent	4.10, 385.00	1.74	.141	.033
Block * AOI	2.17, 203.56	26.44*	<.001	.204
Block * AOI * Agent	4.33, 407.02	0.98	.423	.019
Trial Type * AOI	1.88, 176.34	3647.24*	<.001	.973
Trial Type * AOI * Agent	3.75, 352.68	0.50	.725	.010
Block * Trial Type * AOI	4.12, 386.83	12.36*	<.001	.107
Block * Trial Type * AOI * Agent	8.23, 772.98	1.18	.311	.022

*Note*. Results from RM ANOVA for time in milliseconds spent looking at the AOIs during the Event Phase with agent (Human Hand, Humanoid Hand, Tripod Gripper Hand) as the between-subjects factor and AOI (Nontarget, Center, Target), trial type (congruent, incongruent, control), and block (Block 1, Block 2, Block 3) as the within-subject factors. Mauchly’s Test of Sphericity indicated that the assumption of sphericity was violated; therefore, the corrected results using the Greenhouse-Geisser adjustment are reported. Asterisks denote a statistically significant result at *p* < .05.

**Table 9:** Descriptive statistics for perceived traits.

Variable	Agent Condition	Mean	Standard Error	95% CI [LL, UL]
Anthropomorphism	Human Hand	3.11	0.12	[2.86, 3.36]
	Humanoid Hand	2.13	0.14	[1.84, 2.41]
	Tripod Gripper Hand	2.07	0.15	[1.77, 2.38]
Animacy	Human Hand	2.99	0.13	[2.73, 3.25]
	Humanoid Hand	2.64	0.14	[2.37, 2.92]
	Tripod Gripper Hand	2.64	0.14	[2.36, 2.93]
Likability	Human Hand	2.94	0.16	[2.61, 3.27]
	Humanoid Hand	3.13	0.14	[2.84, 3.42]
	Tripod Gripper Hand	2.67	0.14	[2.39, 2.96]
Intelligence	Human Hand	3.31	0.13	[3.03, 3.58]
	Humanoid Hand	3.11	0.12	[2.88, 3.35]
	Tripod Gripper Hand	2.91	0.14	[2.63, 3.19]
Trust	Human Hand	3.48	0.16	[3.15, 3.81]
	Humanoid Hand	3.92	0.15	[3.63, 4.22]
	Tripod Gripper Hand	3.40	0.15	[3.09, 3.71]

*Note*. Godspeed subscale composite means could range from 1 to 5, with a higher score indicating a greater perception of that trait. The Trust in Automation composite score could range from 1 to 7, with a higher score indicating more perceived trust in the agent. CI stands for confidence interval. LL stands for Lower Limit. UL stands for Upper Limit.

**Table 10:** Unstandardized regression coefficients predicting mediators and trust.

Predictor to Outcome	B	SE	t	p	95% CI [LL, UL]
Agent Condition to Mediators (a-paths)					
HH to Anthropomorphism	−0.98	0.20	−5.01*	<.001	[−1.37, −0.59]
TGH to Anthropomorphism	−1.04	0.20	−5.31*	<.001	[−1.42, −0.65]
HH to Animacy	−0.35	0.19	−1.82	.07	[−0.72, 0.03]
TGH to Animacy	−0.34	0.19	−1.80	.07	[−0.72, 0.03]
HH to Likability	0.19	0.21	0.89	.38	[−0.23, 0.61]
TGH to Likability	−0.26	0.21	−1.25	.21	[−0.68, 0.15]
HH to Intelligence	−0.19	0.18	−1.05	.30	[−0.55, 0.17]
TGH to Intelligence	−0.39	0.18	−2.15*	.03	[−0.75, −0.03]

Mediators to Trust (b-paths)					
Anthropomorphism to Trust	−0.16	0.11	−1.47	.14	[−0.38, 0.06]
Animacy to Trust	0.14	0.12	1.16	.25	[−0.10, 0.37]
Likability to Trust	0.48	0.09	5.64*	<.001	[0.31, 0.66]
Intelligence to Trust	0.37	0.10	3.68*	<.001	[0.17, 0.58]

Total Effects (c-paths)					
Total Effects					
HH to Trust	0.45	0.22	2.06*	.04	[0.02, 0.88]
TGH to Trust	−0.08	0.22	−0.35	.72	[−0.51, 0.35]

Direct Effects (c’ paths)					
HH to Trust	0.32	0.19	1.67	.09	[−0.06, 0.69]
TGH to Trust	0.08	0.19	0.40	.69	[−0.30, 0.45]

*Note*. Human Hand acts as the reference group for the agent conditions. Unstandardized coefficients are reported.

An asterisk (*) indicates a statistically significant path (p < .05).

CI = confidence interval; LL = lower limit; UL = upper limit. HH = Humanoid Hand; TGH = Tripod Gripper Hand.

**Table 11:** Indirect paths from agent condition to trust through mediators.

Indirect Path	B	SE	95% CI [LL, UL]
HH to Anthropomorphism to Trust	0.16	0.12	[−0.07, 0.39]
TGH to Anthropomorphism to Trust	0.17	0.12	[−0.07, 0.42]
HH to Animacy to Trust	−0.05	0.06	[−0.18, 0.04]
TGH to Animacy to Trust	−0.05	0.06	[−0.18, 0.05]
HH to Likability to Trust	0.09	0.10	[−0.11, 0.29]
TGH to Likability to Trust	−0.13	0.11	[−0.38, 0.05]
HH to Intelligence to Trust	−0.07	0.07	[−0.24, 0.05]
TGH to Intelligence to Trust	−0.15*	0.08	[−0.33, −0.01]

*Note*. 95% confidence intervals are based on percentile bootstrapping (5000 samples).

An asterisk (*) indicates a significant indirect effect in which its confidence interval does not include zero.

HH = Humanoid Hand; TGH = Tripod Gripper Hand.

## Appendix A. Supplementary data

Supplementary data to this article can be found online at https://doi.org/10.1016/j.chbah.2026.100356.
