# Supplementary material for: Humans vs. Robots: Utilization of pointing cues and the mediators of trust in nonverbal communication
Source: Comput Hum Behav Artif Hum. Author manuscript; Available in PMC 2026 Oct 1. (PMC13626022; doi:10.1016/j.chbah.2026.100356)
Supplement: Supplementary file 1 [file NIHMS2209974-supplement-1.docx]

**Appendix A**

**Scales Used**

**Appendix A1**

*Godspeed Questionnaire* (Bartneck et al., 2008)

| Anthropomorphism | | | | | | |
| --- | --- | --- | --- | --- | --- | --- |
| Fake | 1 | 2 | 3 | 4 | 5 | Natural |
| Machinelike | 1 | 2 | 3 | 4 | 5 | Humanlike |
| Unconscious | 1 | 2 | 3 | 4 | 5 | Conscious |
| Artificial | 1 | 2 | 3 | 4 | 5 | Lifelike |
| Moving Rigidly | 1 | 2 | 3 | 4 | 5 | Moving elegantly |
| Animacy | | | | | | |
| Dead | 1 | 2 | 3 | 4 | 5 | Alive |
| Stagnant | 1 | 2 | 3 | 4 | 5 | Lively |
| Mechanical | 1 | 2 | 3 | 4 | 5 | Organic |
| Artificial | 1 | 2 | 3 | 4 | 5 | Lifelike |
| Inert | 1 | 2 | 3 | 4 | 5 | Interactive |
| Apathetic | 1 | 2 | 3 | 4 | 5 | Responsive |
| Likeability | | | | | | |
| Dislike | 1 | 2 | 3 | 4 | 5 | Like |
| Unfriendly | 1 | 2 | 3 | 4 | 5 | Friendly |
| Unkind | 1 | 2 | 3 | 4 | 5 | Kind |
| Unpleasant | 1 | 2 | 3 | 4 | 5 | Pleasant |
| Awful | 1 | 2 | 3 | 4 | 5 | Nice |
| Perceived Intelligence | | | | | | |
| Incompetent | 1 | 2 | 3 | 4 | 5 | Competent |
| Ignorant | 1 | 2 | 3 | 4 | 5 | Knowledgeable |
| Irresponsible | 1 | 2 | 3 | 4 | 5 | Responsible |
| Unintelligent | 1 | 2 | 3 | 4 | 5 | Intelligent |
| Foolish | 1 | 2 | 3 | 4 | 5 | Sensible |

**Appendix A2**

*Modified Trust in Automation Scale* (Jian et al., 2000)

**1. The arm is deceptive**

| Not at all | 1 | 2 | 3 | 4 | 5 | 6 | 7 | Extremely |
| --- | --- | --- | --- | --- | --- | --- | --- | --- |
|  |  |  |  |  |  |  |  |  |

**2. The arm behaves in an underhanded manner**

| Not at all | 1 | 2 | 3 | 4 | 5 | 6 | 7 | Extremely |
| --- | --- | --- | --- | --- | --- | --- | --- | --- |

**3. I am suspicious of the arm’s intent, action, or outputs**

| Not at all | 1 | 2 | 3 | 4 | 5 | 6 | 7 | Extremely |
| --- | --- | --- | --- | --- | --- | --- | --- | --- |

**4. I am wary of the arm**

| Not at all | 1 | 2 | 3 | 4 | 5 | 6 | 7 | Extremely |
| --- | --- | --- | --- | --- | --- | --- | --- | --- |

**5. The arm’s actions will have a harmful or injurious outcome**

| Not at all | 1 | 2 | 3 | 4 | 5 | 6 | 7 | Extremely |
| --- | --- | --- | --- | --- | --- | --- | --- | --- |

**6. I am confident in the arm**

| Not at all | 1 | 2 | 3 | 4 | 5 | 6 | 7 | Extremely |
| --- | --- | --- | --- | --- | --- | --- | --- | --- |

**7. The arm provides security**

| Not at all | 1 | 2 | 3 | 4 | 5 | 6 | 7 | Extremely |
| --- | --- | --- | --- | --- | --- | --- | --- | --- |

**8. The arm has integrity**

| Not at all | 1 | 2 | 3 | 4 | 5 | 6 | 7 | Extremely |
| --- | --- | --- | --- | --- | --- | --- | --- | --- |

**9. The arm is dependable**

| Not at all | 1 | 2 | 3 | 4 | 5 | 6 | 7 | Extremely |
| --- | --- | --- | --- | --- | --- | --- | --- | --- |

**10. The arm is reliable**

| Not at all | 1 | 2 | 3 | 4 | 5 | 6 | 7 | Extremely |
| --- | --- | --- | --- | --- | --- | --- | --- | --- |

**11. I can trust the arm**

| Not at all | 1 | 2 | 3 | 4 | 5 | 6 | 7 | Extremely |
| --- | --- | --- | --- | --- | --- | --- | --- | --- |

**12. I am familiar with the arm**

| Not at all | 1 | 2 | 3 | 4 | 5 | 6 | 7 | Extremely |
| --- | --- | --- | --- | --- | --- | --- | --- | --- |

**Appendix B**

**Preliminary Analyses for Gender Differences**

**Appendix B1**

*RM ANOVA Test of Within-Subject Effects with Gender Included for Action Phase*

| Source | Type III Sum of Squares | df | Mean Square | F | Sig. | Partial Eta Squared |
| --- | --- | --- | --- | --- | --- | --- |
| Trial Type | 13854.45 | 1.00 | 13854.45 | 0.17 | 0.68 | 0.00 |
| Trial Type * Agent | 336425.37 | 2.00 | 168212.69 | 2.05 | 0.13 | 0.04 |
| Trial Type * Gender | 245898.76 | 1.00 | 245898.76 | 2.99 | 0.09 | 0.03 |
| Trial Type * Agent * Gender | 70262.53 | 2.00 | 35131.26 | 0.43 | 0.65 | 0.01 |
| AOI | 8211638.84 | 100.00 | 82116.39 |  |  |  |
| AOI * Agent | 9843101402.08 | 1.13 | 8725859234.96 | 2078.05 | 0.000 | 0.95 |
| AOI * Gender | 21483038.68 | 2.26 | 9522302.16 | 2.27 | 0.10 | 0.04 |
| AOI * Agent * Gender | 1484714.67 | 1.13 | 1316191.99 | 0.31 | 0.60 | 0.00 |
| Trial Type * AOI | 20819966.45 | 2.26 | 9228397.08 | 2.20 | 0.11 | 0.04 |
| Trial Type * AOI * Agent | 473669265.57 | 112.80 | 4199053.90 |  |  |  |
| Trial Type * AOI * Gender | 170429896.18 | 1.10 | 154442021.76 | 152.27 | 0.000 | 0.60 |
| Trial Type | 11086479.28 | 2.21 | 5023233.34 | 4.95 | 0.01 | 0.09 |
| Trial Type * Agent | 1583966.62 | 1.10 | 1435376.14 | 1.42 | 0.24 | 0.01 |
| Trial Type * Gender | 4231274.28 | 2.21 | 1917171.14 | 1.89 | 0.15 | 0.04 |
| Trial Type * Agent * Gender | 111928924.77 | 110.35 | 1014289.74 |  |  |  |

*Note.* A 3 (Agent: HUH, HH, TGH) × 3 (AOI: center, nontarget, target) × 2 (Trial Type: cued, control) × 2 (Gender: female, male) mixed-model repeated measures analysis of variance (RM ANOVA) was performed on average dwell times (in milliseconds) during the Action phase of the test trials. Agent condition and gender were treated as between-subjects factors, while AOI and trial type were within-subjects factors.

**Appendix B2**

*RM ANOVA Test of Within-Subject Effects with Gender Included for Prediction Phase*

| Source | Type III Sum of Squares | df | Mean Square | F | Sig. | Partial Eta Squared |
| --- | --- | --- | --- | --- | --- | --- |
| Trial Type | 29057.82 | 1.00 | 29057.82 | 1.96 | 0.17 | 0.02 |
| Trial Type * Agent | 97753.61 | 2.00 | 48876.80 | 3.29 | 0.04 | 0.06 |
| Trial Type * Gender | 991.06 | 1.00 | 991.06 | 0.07 | 0.80 | 0.00 |
| Trial Type * Agent * Gender | 19622.66 | 2.00 | 9811.33 | 0.66 | 0.52 | 0.01 |
| AOI | 191004032.66 | 1.12 | 171316605.93 | 209.30 | 0.000 | 0.68 |
| AOI * Agent | 2722494.47 | 2.23 | 1220938.91 | 1.49 | 0.23 | 0.03 |
| AOI * Gender | 425210.90 | 1.12 | 381382.99 | 0.47 | 0.52 | 0.01 |
| AOI * Agent * Gender | 2012615.95 | 2.23 | 902584.44 | 1.10 | 0.34 | 0.02 |
| Trial Type * AOI | 3268311.27 | 1.32 | 2468343.54 | 18.90 | 0.000 | 0.16 |
| Trial Type * AOI * Agent | 1642042.81 | 2.65 | 620064.22 | 4.75 | 0.01 | 0.09 |
| Trial Type * AOI * Gender | 149896.80 | 1.32 | 113207.33 | 0.87 | 0.38 | 0.01 |
| Trial Type * AOI * Agent * Gender | 477274.83 | 2.65 | 180227.36 | 1.38 | 0.25 | 0.03 |

*Note.* A 3 (Agent: HUH, HH, TGH) × 3 (AOI: center, nontarget, target) × 2 (Trial Type: cued, control) × 2 (Gender: female, male) mixed-model repeated measures analysis of variance (RM ANOVA) was performed on average dwell times (in milliseconds) during the Action phase of the test trials. Agent condition and gender were treated as between-subjects factors, while AOI and trial type were within-subjects factors.
